# Supplementary material for: Three-dimensional covalent organic frameworks with nia nets for efficient separation of benzene/cyclohexane mixtures
Source: Nat Commun. 2024 Jan 27;15:813. doi: 10.1038/s41467-024-45005-8 (PMC10821887; doi:10.1038/s41467-024-45005-8)
Supplement: Supplementary file 1 — Supplementary Information [file 41467_2024_45005_MOESM1_ESM.pdf]

Supplementary Information

Three-Dimensional Covalent Organic Frameworks with nia Nets for Efficient Separation of Benzene/Cyclohexane Mixtures

Jianhong Chang,[†] Fengqian Chen,[†] Hui Li, Jinquan Suo, Haorui Zheng, Jie Zhang, Zitao Wang, Liangkui Zhu, Valentin Valtchev, Shilun Qiu, and Qianrong Fang**

Supplementary Methods

1. Chemical syntheses

1.1. Synthesis of 2,3,6,7,14,15-hexa(4′-formylphenyl)triptycene (HFPTP-H)[1]

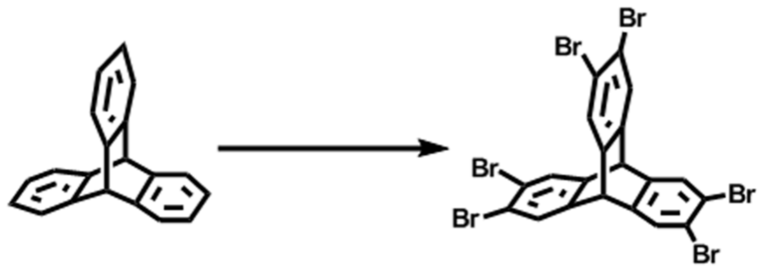

(1) Synthesis of 2,3,6,7,14,15-hexabromotriptycene

Triptycene (1.00 g, 3.9 mmol) and iron powder (80.0 mg, 1.45 mmol) were dissolved in 1,2-dichloroethane (60.0 mL) and then bromine (1.32 mL, 25.7 mmol) was added slowly to the flask. The mixture was circularly refluxed for 6 h. All the solvent was removed under reduced pressure after the temperature of the reaction was cooled to 25 °C. The leftover was loaded with a column (silica, $CHCl_3$) to give solid, which was recrystallized from $CHCl_3$ to give the pure product as colorless, needle-like crystals: (2.24 g, 3.1 mmol, 79%), m.p. > 350 °C; ^{1}H NMR (500 MHz, $CDCl_3$, 300 K): δ (ppm) 7.62 (s, 6 H), 5.24 (s, 2 H).

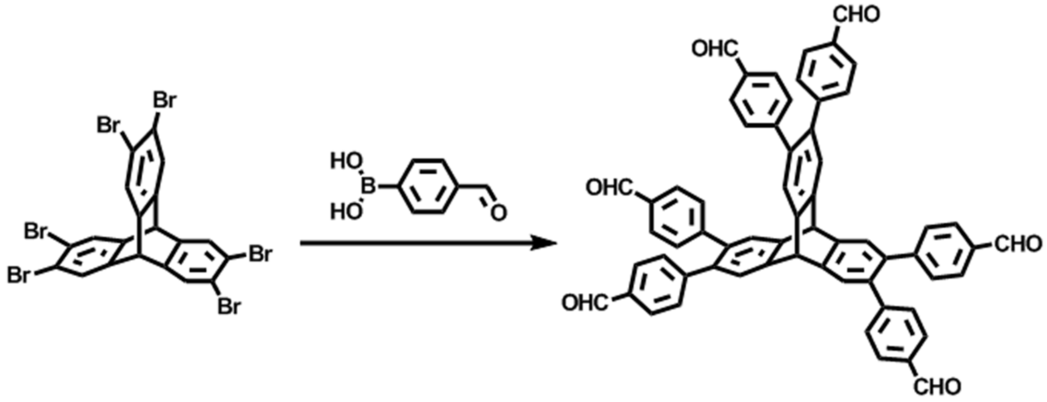

(2) Synthesis of HFPTP-H

2,3,6,7,14,15-hexabromotriptycene (500.0 mg, 0.69 mmol), Cs_2CO_3 (2.90 g, 8.9 mmol), $Pd(PPh_3)_4$ (0.24 g, 0.2 mmol) and (4-formylphenyl)boronic acid (1.33 g, 8.9 mmol) were dissolved in anhydrous THF (50.0 mL). Then the mixture was heated and stirred at 65 °C with an argon atmosphere for 18 h. The solvent was cleared under reduced pressure and the rest mixture was

dissolved in CH_2Cl_2 (100.0 mL). The rough product was rinsed sequentially [saturated $NaHCO_3$ (100.0 mL), deionized H_2O (100.0 mL) and brine (100.0 mL)]. $MgSO_4$ was used to dry and the organic phase was filtered. The solvent was cleared under vacuo and the unshaped product was depurated by column chromatography with silica gel (CH_2Cl_2/methanol, 25:1, v/v) and gained pure product as white crystals (426.0 mg, 0.48 mmol, 70%), m.p. > 300 °C. Rf = 0.55 (CH_2Cl_2/methanol, 20:1, v/v). ^1H NMR (400 MHz, $CDCl_3$): δ = 9.96 (s, 6 H), 7.72 (d, J = 8.0 Hz, 12 H), 7.61 (s, 6 H), 7.24 (d, J = 8.0 Hz, 12 H), 5.78 (s, 2 H) ppm.

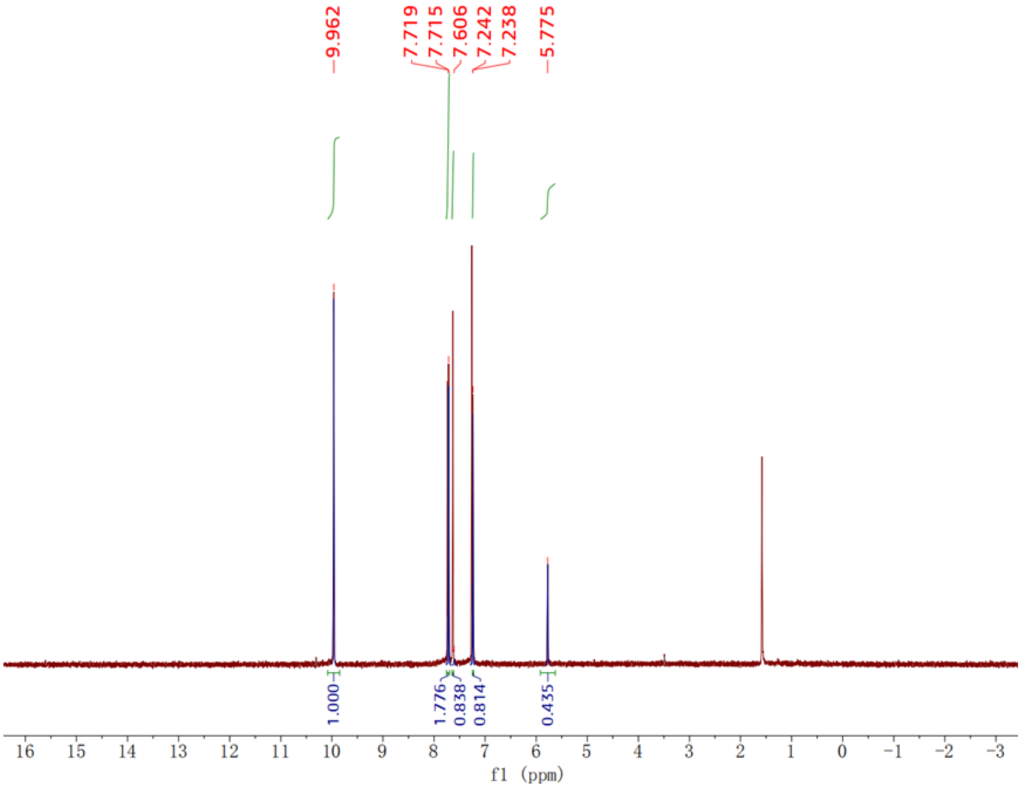

Supplementary Figure 1. ^1H NMR spectra of HFPTP-H. (Frequency: 400 MHz, solvent: $CDCl_3$)

1.2. Synthesis of 2,3,6,7,14,15-hexa(3'-fluorine-4′-formylphenyl)triptycene (HFPTP-F)[1]

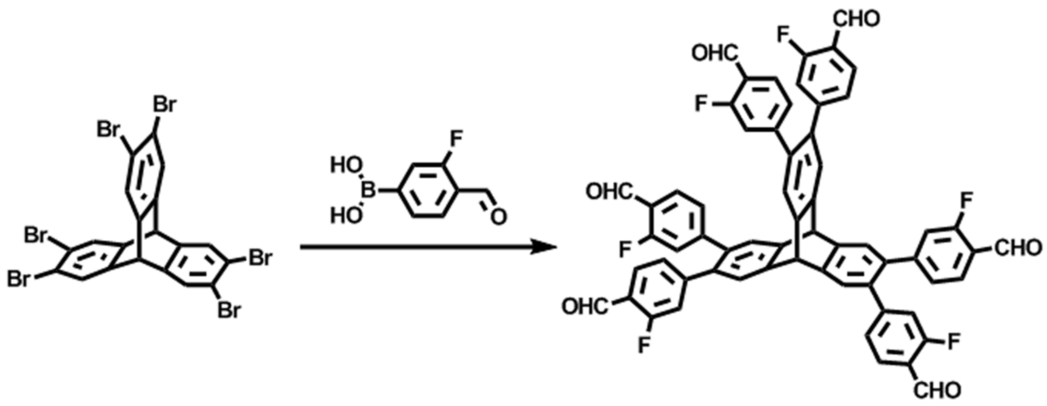

(1) Synthesis of HFPTP-F

2,3,6,7,14,15-hexabromotriptycene (500.0 mg, 0.69 mmol), Cs_2CO_3 (2.90 g, 8.9 mmol), $Pd(PPh_3)_4$ (0.24 g, 0.2 mmol) and (3-Fluoro-4-formylphenyl) boronic acid (1.49 g, 8.9 mmol) were dissolved in anhydrous THF (50.0 mL). Then it was heated and stirred at 65 °C in an argon atmosphere for 18 h. The solvent was eliminated at reduced pressure and the residual compound was dissolved in CH_2Cl_2 (100.0 mL). The rough product was laved sequentially with saturated $NaHCO_3$ (100.0 mL), deionized H_2O (100.0 mL) and brine (100.0 mL). The organic phase was dried with $MgSO_4$ and filtered. The solvent was removed in vacuo and the crude product was purified by column chromatography with silica gel (CH_2Cl_2/methanol, 50:1, v/v) and obtained pure product as white crystals (364.8 mg, 0.37 mmol, 53%), m.p. > 300 °C. Rf = 0.55 (CH_2Cl_2/methanol, 50:1, v/v). ^1H NMR (400 MHz, $CDCl_3$): δ (ppm): 10.31 (s, 6 H), 7.71-7.74 (m, J = 7.88, 6 H), 7.60 (s, 6 H), 6.91-6.97 (m, J = 8.04, 12 H), 5.77 (s, 2 H).

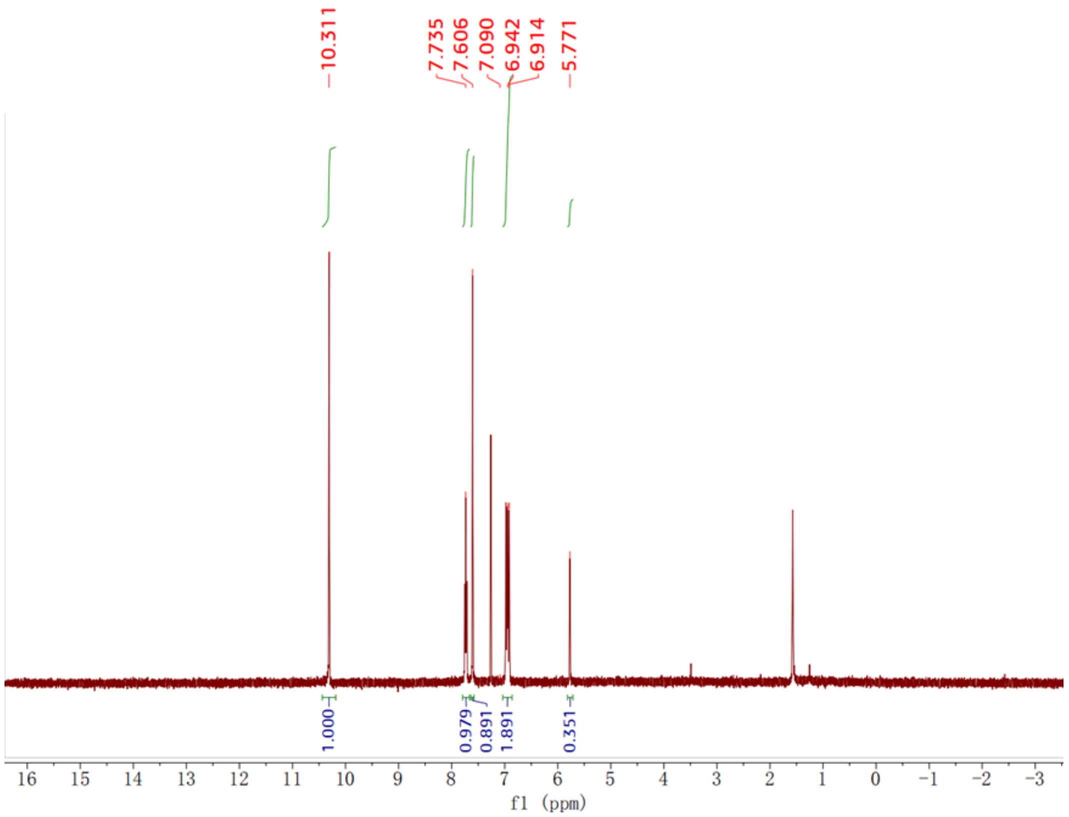

Supplementary Figure 2. ^{1}H NMR spectra of HFPTP-F. (Frequency: 400 MHz, solvent: CDCl3)

1.3. Synthesis of 2,3,6,7,10,11-hexa(4'-aminophenyl) trimethylene (HAPTM)[2]

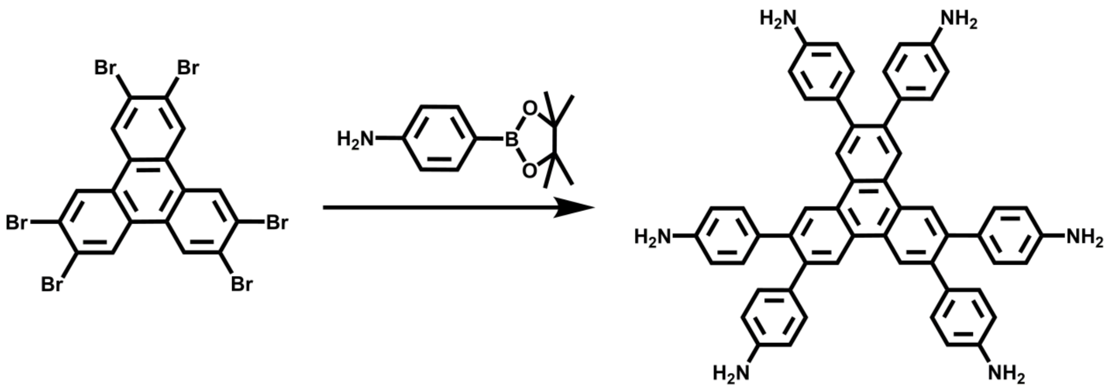

To the mixture of boronic ester (700 mg, 3.20 mmol) and $PdCl_2(PPh_3)_2$ (75 mg,0.11 mmol) in 1,4-dioxane was added suspension of 2,3,6,7,10,11-hexabromotriphenylene (300 mg, 0.42 mmol) in aqueous solution of K_2CO_3 (707 mg, 5.12 mmol). The mixture was refluxed overnight at 90 ^oC under nitrogen and allowed to cool at room temperature. On addition of water to the reaction mixture, brown solid precipitated out which was filtered, dried and then purified by column chromatography ($CHCl_3$/MeOH, 19:1, v/v) to give the brown crystals in 70% yield. ^{1}H NMR (400 MHz, DMSO) δ

(ppm): 5.06 (s, NH_2, 12 H), 6.49 (d, J = 7.2 MHz, ArH, 12 H), 6.98 (d, J = 7.2 MHz, ArH, 12 H), 8.49 (s, ArH, 6 H).

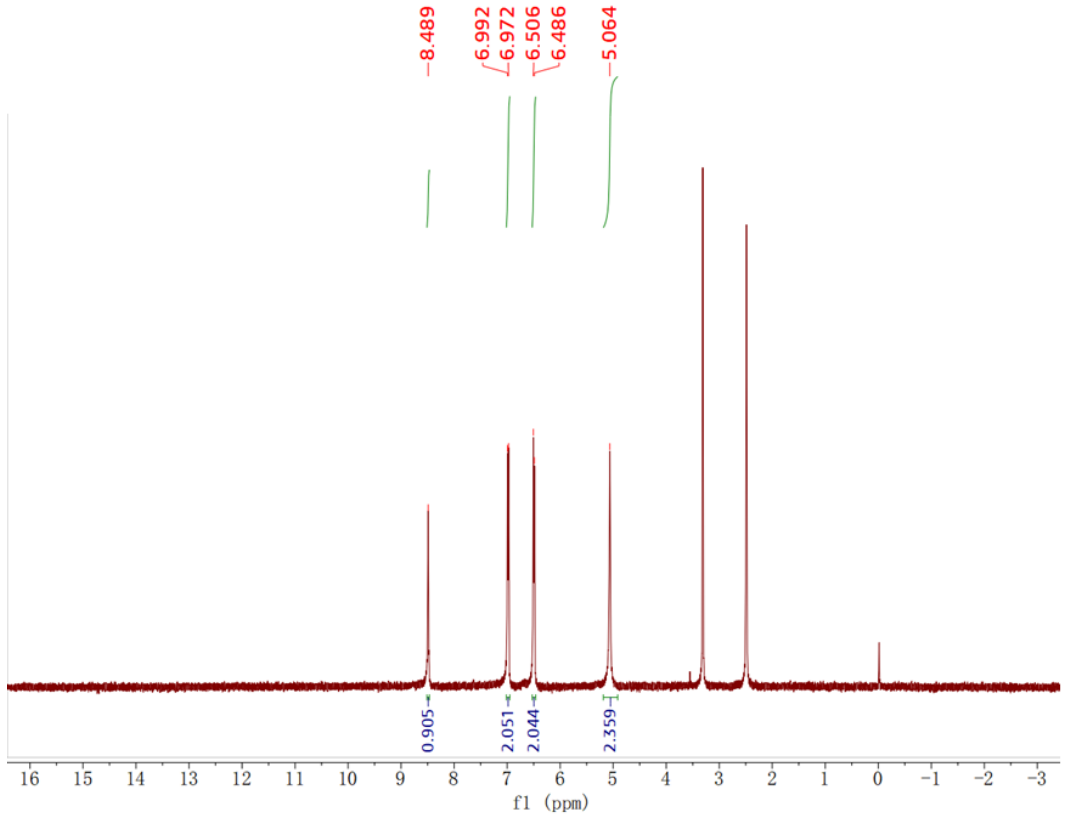

Supplementary Figure 3. ^{1}H NMR spectra of HAPTM. (Frequency: 400 MHz, solvent: DMSO)

1.4. Synthesis of JUC-641 and JUC-642

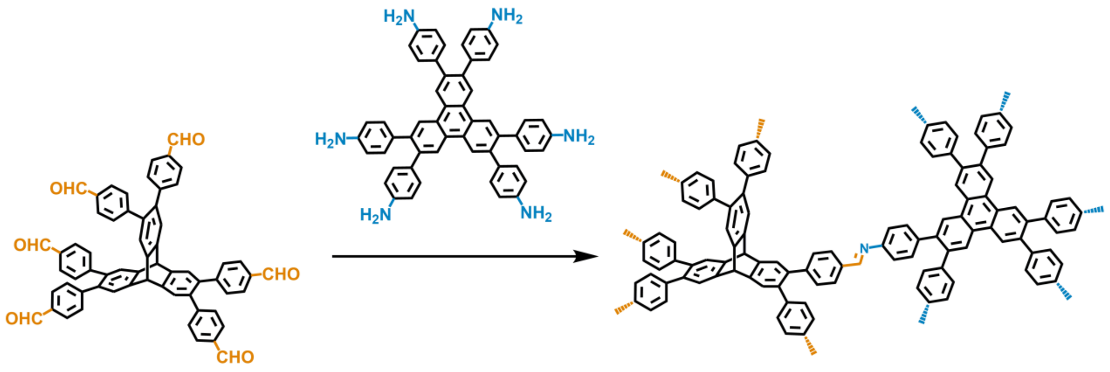

HFPTP-H (0.016 mmol, 14.0 mg) and HAPTM (0.016 mmol, 12.4 mg) were loaded into a Pyrex tube, 2.0 mL 1,4-dioxane and 0.2 mL of acetic acid (6 M) was added. The liquid nitrogen bath was used to freeze the Pyrex tube, which was evacuated to an interior pressure of ca. 19.0 mbar and

flame-sealed, decreasing the whole length by ca. 10.0 cm. The tube was placed in an oven at 120 °C for 7 d after the temperature increasing to room temperature. The resulting precipitate was filtered, exhaustively washed with acetone for 5 times. The obtained powder was immersed in anhydrous n-hexane, and the solvent was exchanged with fresh n-hexane for several times. The sample was then transferred to vacuum chamber and evacuated to 20 mTorr under 65 °C, yielding white powder for N_2 adsorption measurements. Anal. Cald: C: 90.27; H: 4.28; N: 5.45. Found: C: 90.85; H: 4.62; N: 4.53.

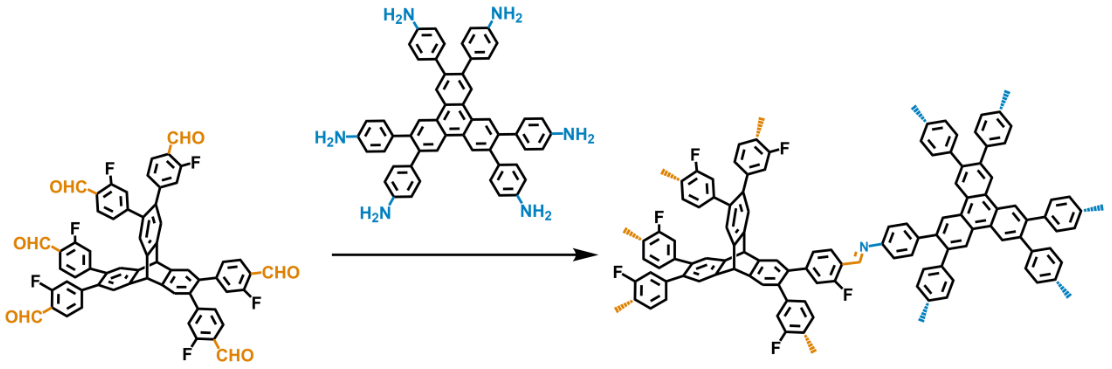

HFPTP-F (0.015 mmol, 15.0 mg) and HAPTM (0.015 mmol, 11.6 mg) were loaded into a Pyrex tube, 1.0 mL 1,4-dioxane and 0.1 mL of acetic acid (6 M) was added. The liquid nitrogen bath was used to freeze the Pyrex tube, which was evacuated to an interior pressure of ca. 19.0 mbar and flame-sealed, decreasing the whole length by ca. 10.0 cm. The tube was placed in an oven at 120 °C for 7 d after the temperature increasing to room temperature. The resulting precipitate was filtered, exhaustively washed with acetone for 5 times. The obtained powder was immersed in anhydrous n-hexane, and the solvent was exchanged with fresh n-hexane for several times. The sample was then transferred to vacuum chamber and evacuated to 20 mTorr under 65 °C, yielding white powder for N_2 adsorption measurements. Anal. Cald: C: 84.36; H: 3.64; N: 5.09; F: 6.91. Found: C: 84.78; H: 3.24; N: 4.97; F: 7.01.

1.5. Calculation of the overall energy of the structure

All periodic density functional calculations were executed using the Gaussian plane-wave computational package CP2K 2023.1.[3] To account for the weak interactions, Grimme's dispersion correction (D3-BJ) was included in each calculation.[4,11] All crystalline structures were firstly optimized using combination of PBE theoretical method and DZVP-MOLOPT-SR-GTH basis set

with cutoff set of 400 Ry to get reasonable geometric conformations. Then, we chose PBE0as theoretical method and TZVP-MOLOPT-GTH as basis set and set the cutoff to 450Ry to calculated single point energies of the DFT optimized structures.[5]

To compare the single point energies of the two structures with different number of atoms in the cell, we set the greatest common divisor of the number of atoms in the two structures as a unit (C_{232} H_{124} N_{12} F_{12}) and process the results to get the energy values correspond to each unit. The generated results show that the energy of nia-net is 71.7 kJ/unit lower than that of htp-net, indicating the nia-net is energy preferred structure.

Supplementary Table 1. Single point energy of two topological nets.

COF	Composition	Energy per cell (kJ)	Energy per unit (kJ)
**htp-F-642**	C464 H248 N24 F24	-9469945.706	-4734972.853
**nia-F-642**	C232 H124 N12 F12	-4735044.526	-4735044.526

Supplementary Figures.

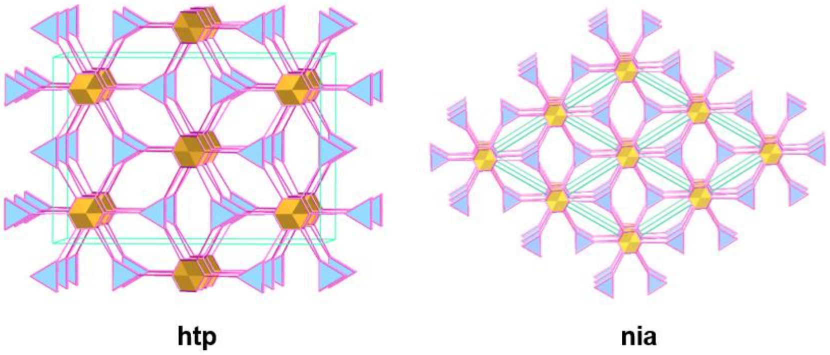

Supplementary Figure 4. Schematic representation of the (6, 6)-connected 3D networks with different topologies: **htp** and **nia**.

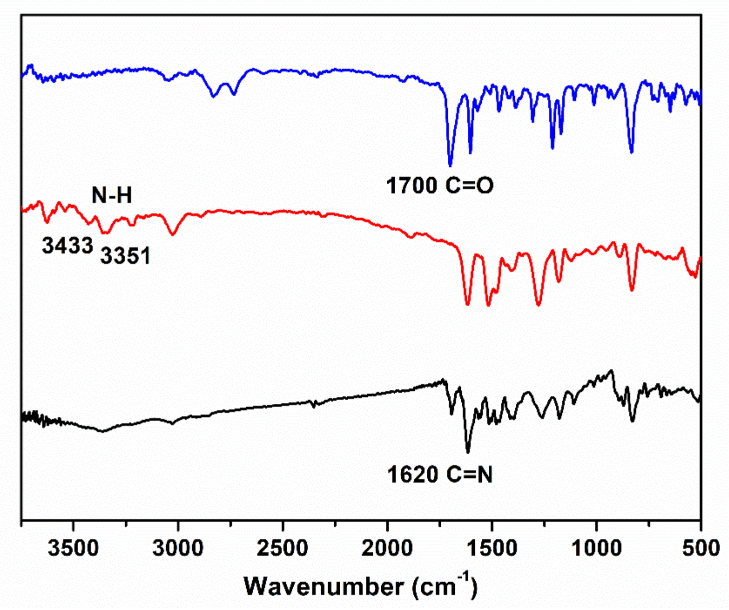

Supplementary Figure 5. FT-IR spectra of HFPTP-H (blue) and HAPTM (red), JUC-641 (black).

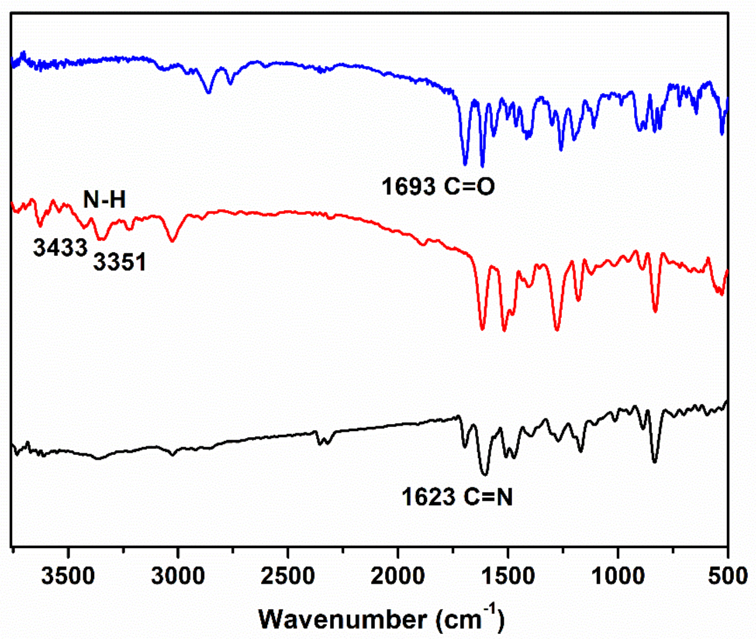

Supplementary Figure 6. FT-IR spectra of HFPTP-F (blue) and HAPTM (red), JUC-642 (black).

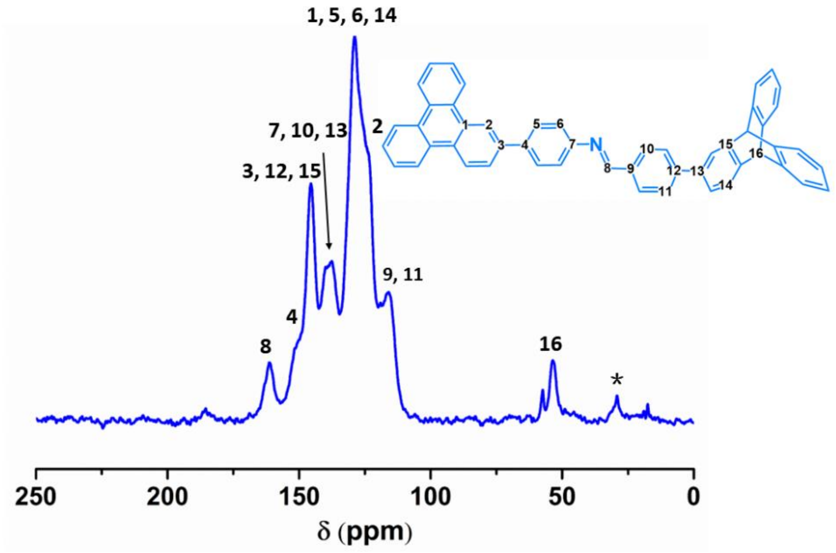

Supplementary Figure 7. Solid state ^{13}C NMR of JUC-641, spinning sidebands are marked by asterisks.

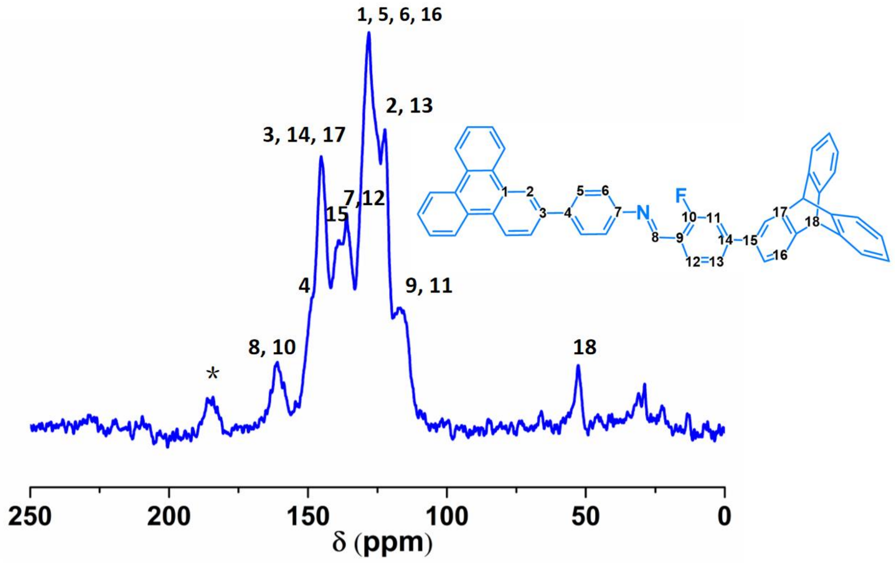

Supplementary Figure 8. Solid state ^{13}C NMR of JUC-642, spinning sidebands are marked by asterisks.

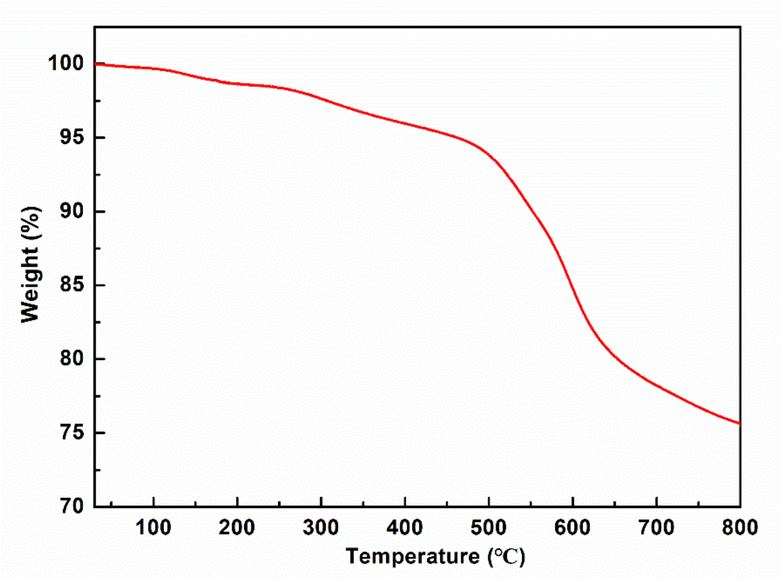

Supplementary Figure 9. TGA curve of JUC-641.

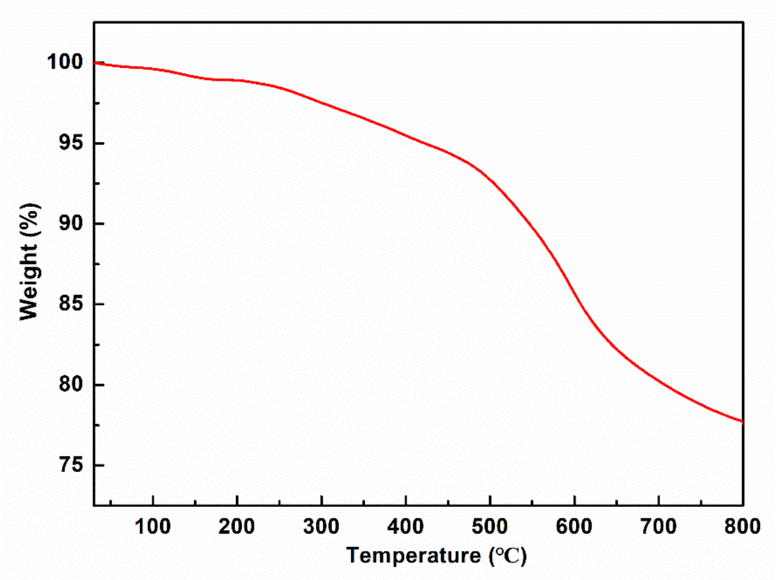

Supplementary Figure 10. TGA curve of JUC-642.

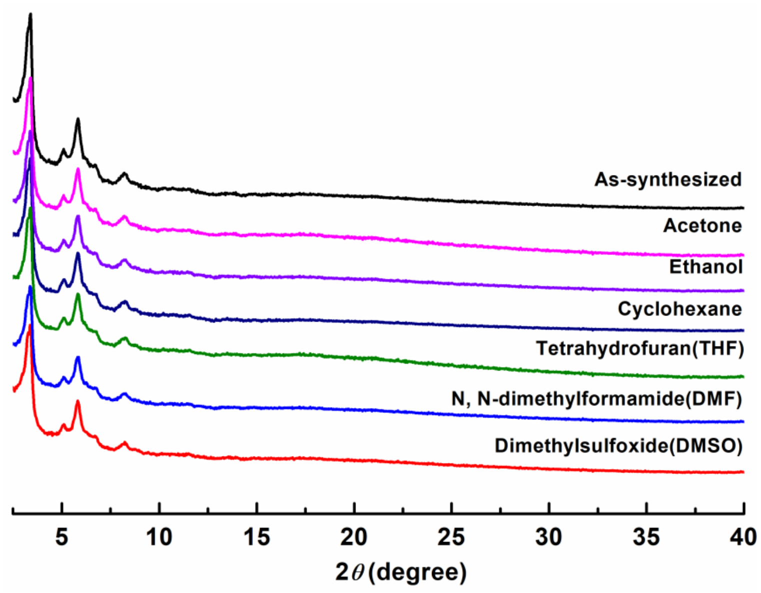

Supplementary Figure 11. PXRD patterns of JUC-641 after the treatment in different organic solvents for 24 h.

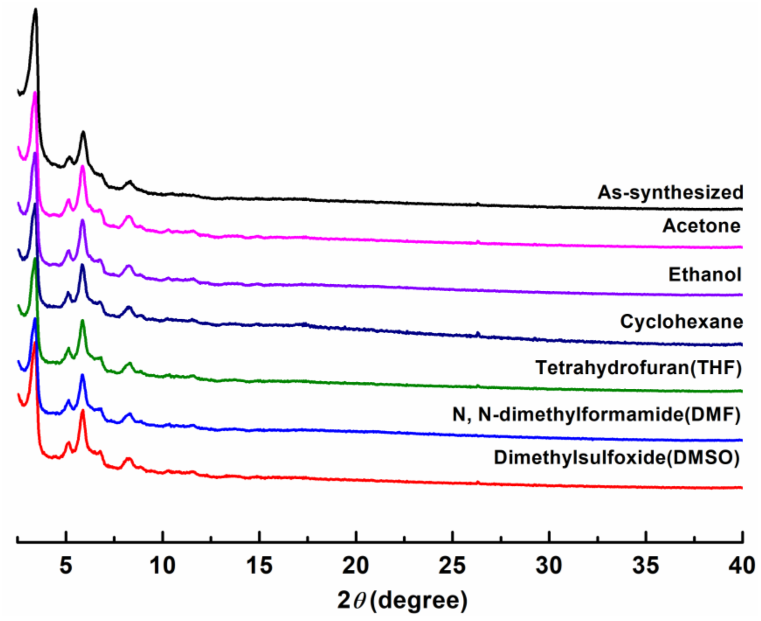

Supplementary Figure 12. PXRD patterns of JUC-642 after the treatment in different organic solvents for 24 h.

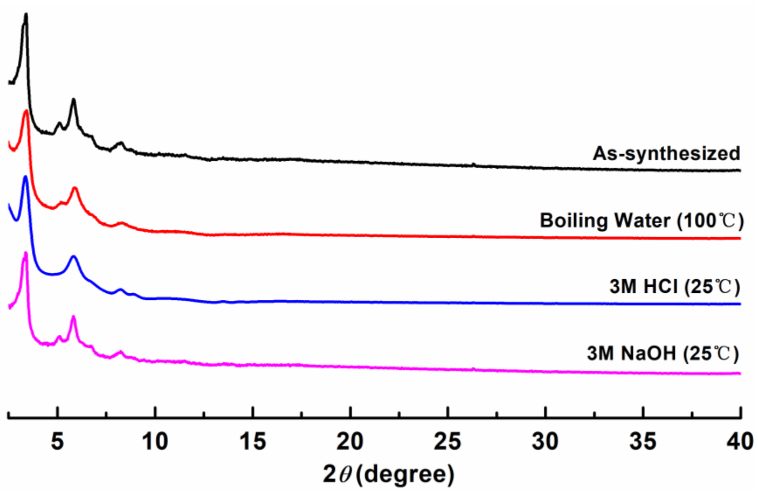

Supplementary Figure 13. PXRD patterns of JUC-641 after the treatment in boiling water (100 °C), strong acid (3 M HCl, 25 °C) and strong base (3 M NaOH, 25 °C) for 24 h.

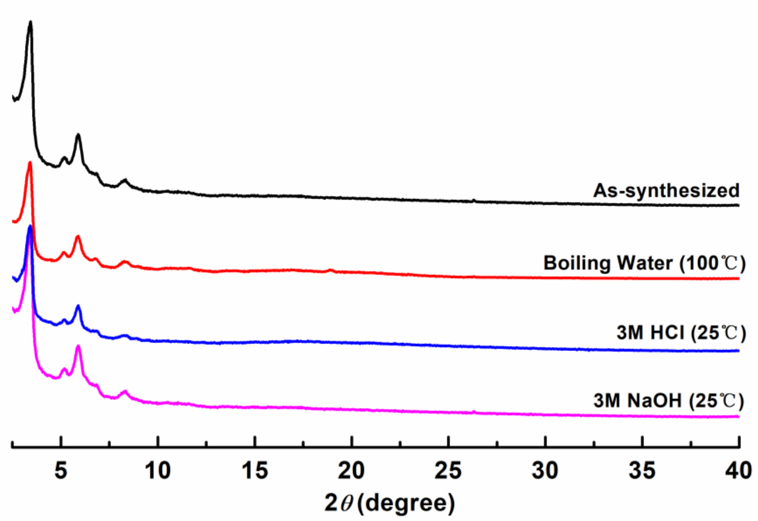

Supplementary Figure 14. PXRD patterns of JUC-642 after the treatment in boiling water (100 °C), strong acid (3 M HCl, 25 °C) and strong base (3 M NaOH, 25 °C) for 24 h.

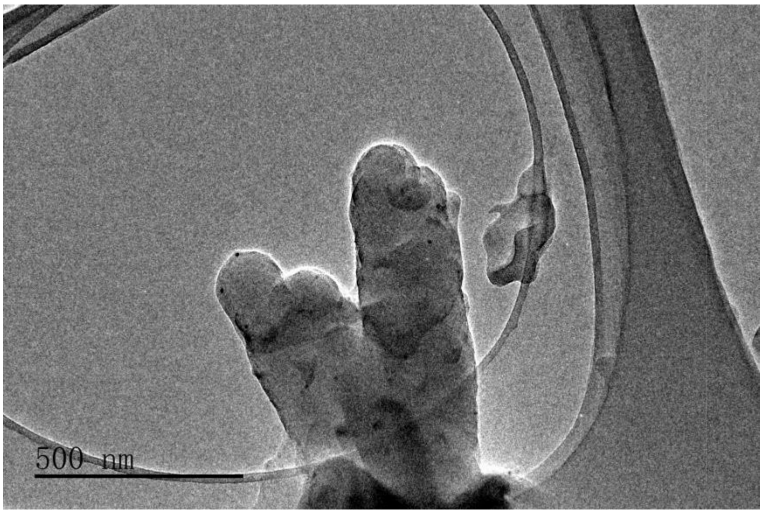

Supplementary Figure 15. TEM image of JUC-641.

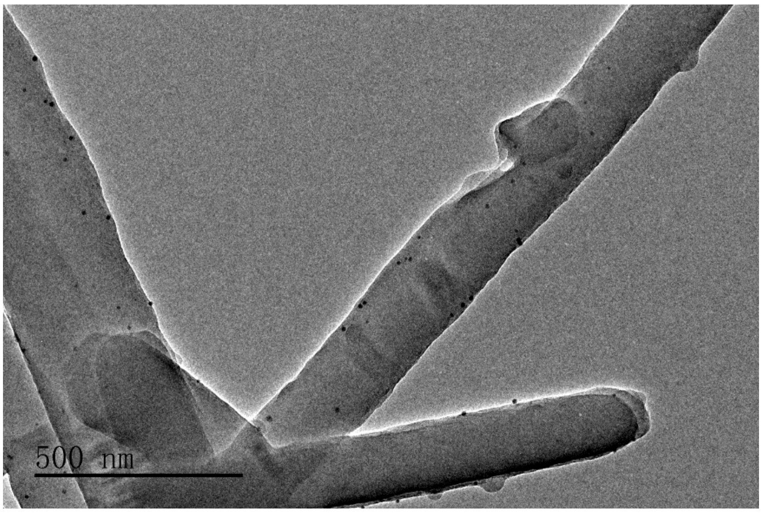

Supplementary Figure 16. TEM image of JUC-642.

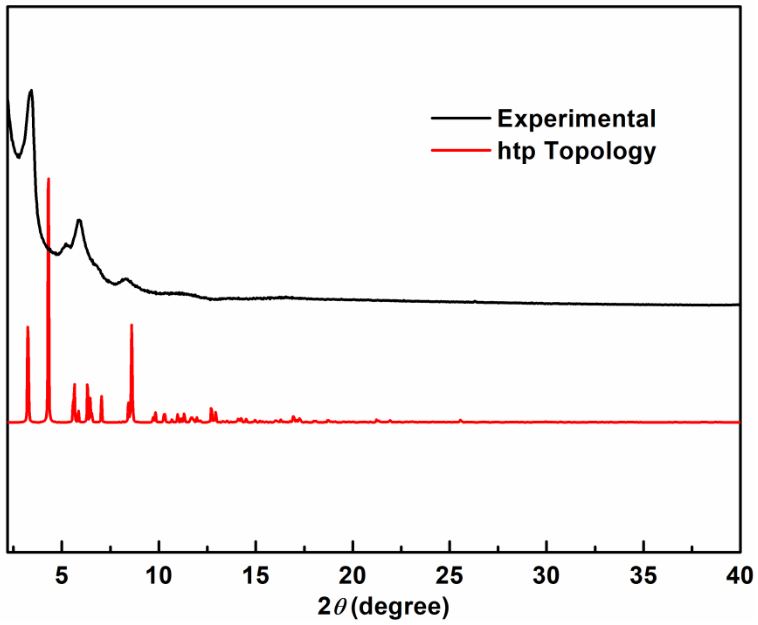

Supplementary Figure 17. Comparison of PXRD patterns for JUC-641: experimental (black), **htp** topology (red).

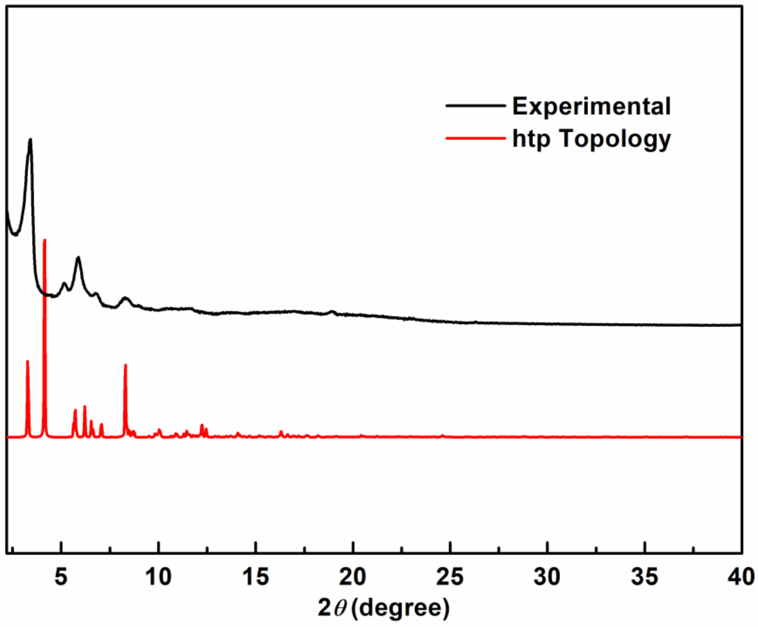

Supplementary Figure 18. Comparison of PXRD patterns for JUC-642: experimental (black), **htp** topology (red).

In the hexagonal crystal system, the formula for calculating the interplanar spacing (d) is:

$$d_{hkl} = \frac{1}{\sqrt{\frac{4}{3}\left(\frac{h^2 + k^2 + hk}{a^2}\right) + \left(\frac{l}{c}\right)^2}}$$

Here, d represents the interplanar spacing, *a* and *c* is the lattice constant, and *h*, *k*, and *l* are the Miller indices of the crystal planes.

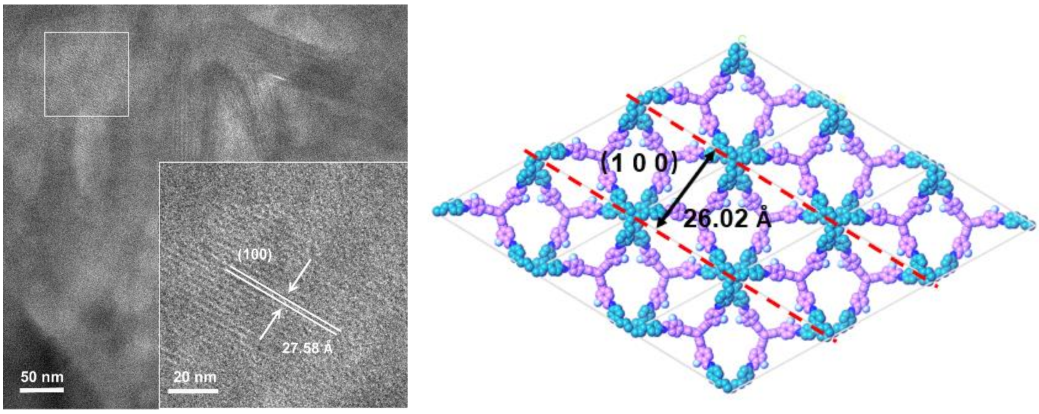

Supplementary Figure 19. HRTEM images and simulated crystal structure of JUC-642 on the (1 0 0) crystal plane.

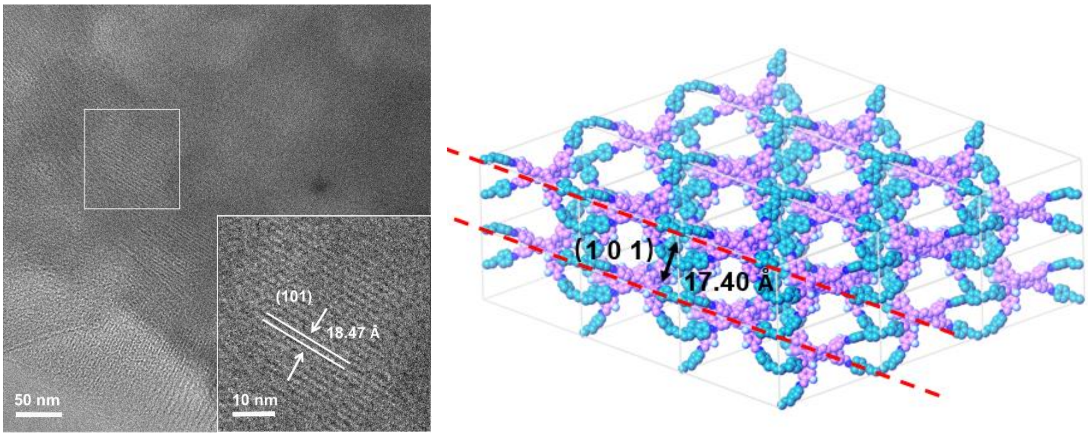

Supplementary Figure 20. HRTEM images and simulated crystal structure of JUC-642 on the (1 0 1) crystal plane.

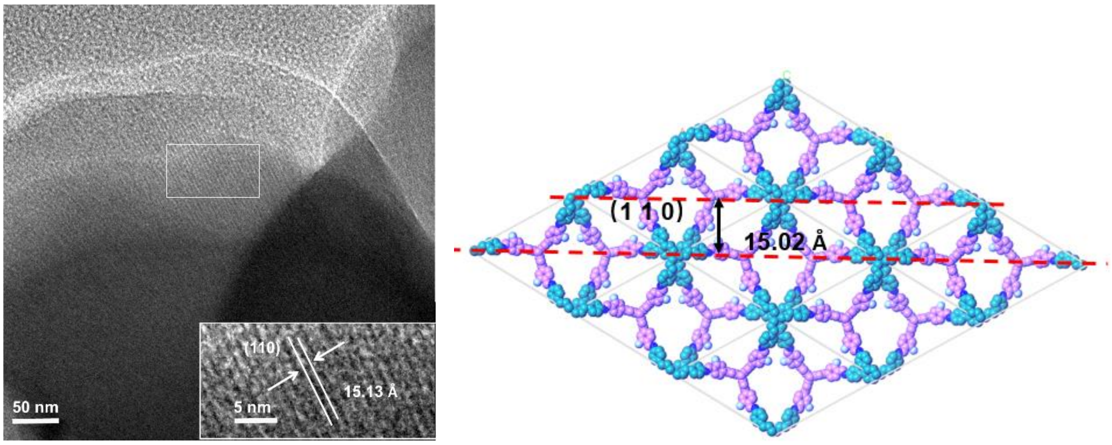

Supplementary Figure 21. HRTEM images and simulated crystal structure of JUC-642 on the (1 1 0) crystal plane.

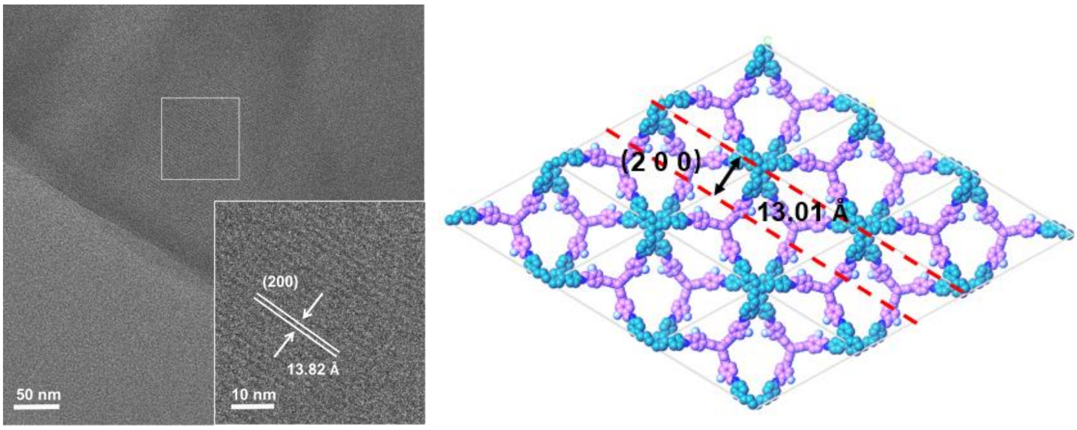

Supplementary Figure 22. HRTEM images and simulated crystal structure of JUC-642 on the (2 0 0) crystal plane.

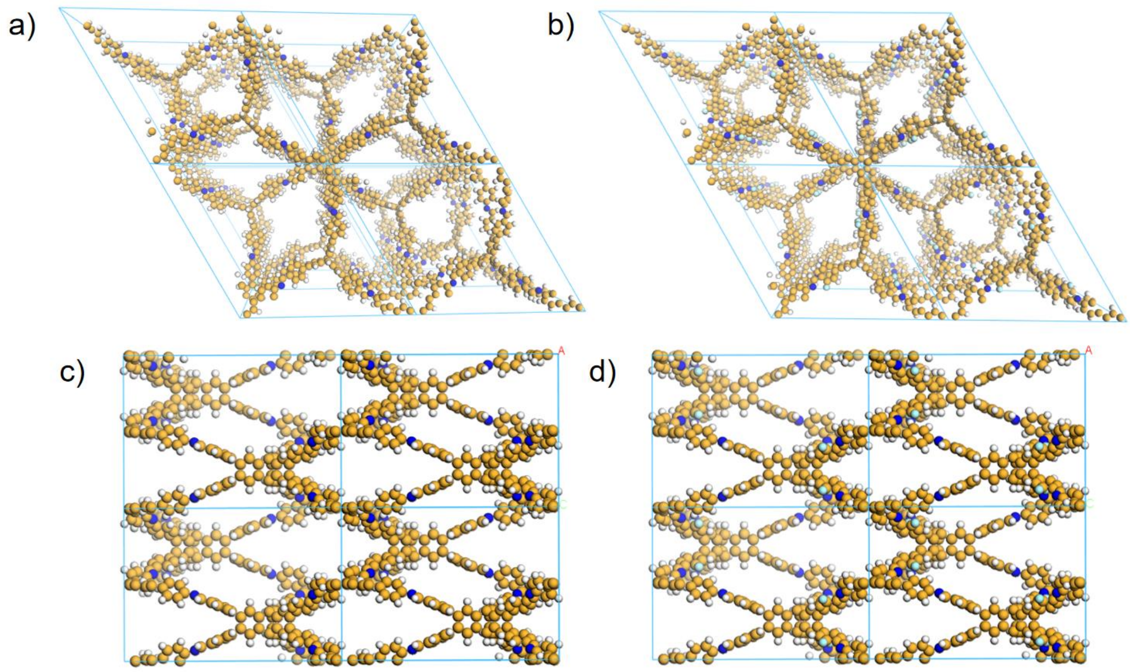

Supplementary Figure 23. Expanded structures of JUC-641 observed along *c* axis (a) and *a* or *b* axis (c). Expanded structures of JUC-642 observed along *c* axis (b) and *a* or *b* axis (d). C, yellow; H, white; F, purple, N, blue.

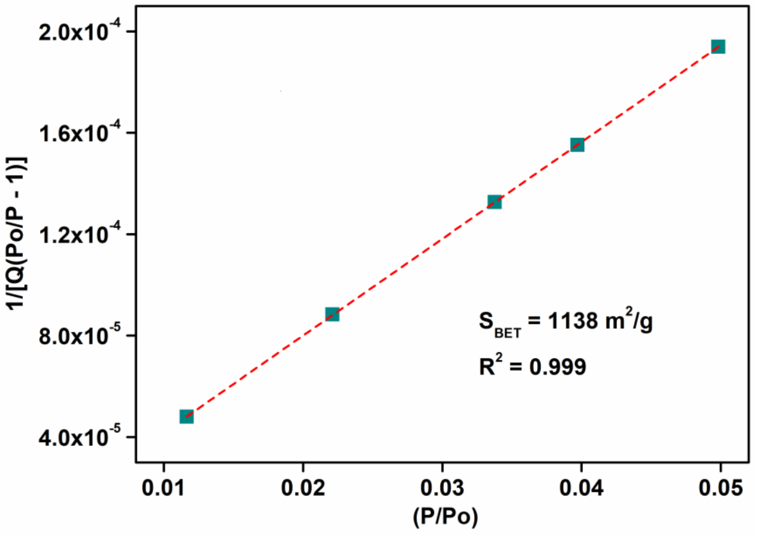

Supplementary Figure 24. BET plot of JUC-641 calculated from N_2 adsorption isotherm at 77 K.

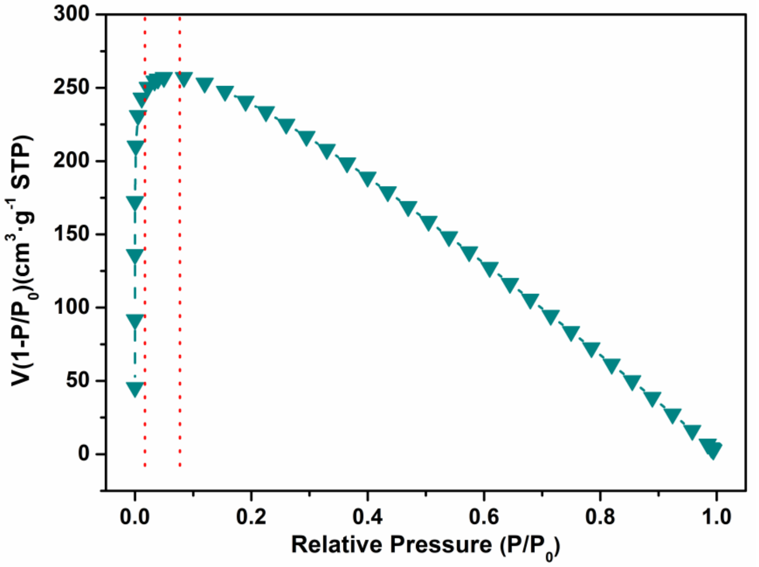

Supplementary Figure 25. Rouquerol BET of JUC-641 calculated from N_2 adsorption isotherm at 77 K.

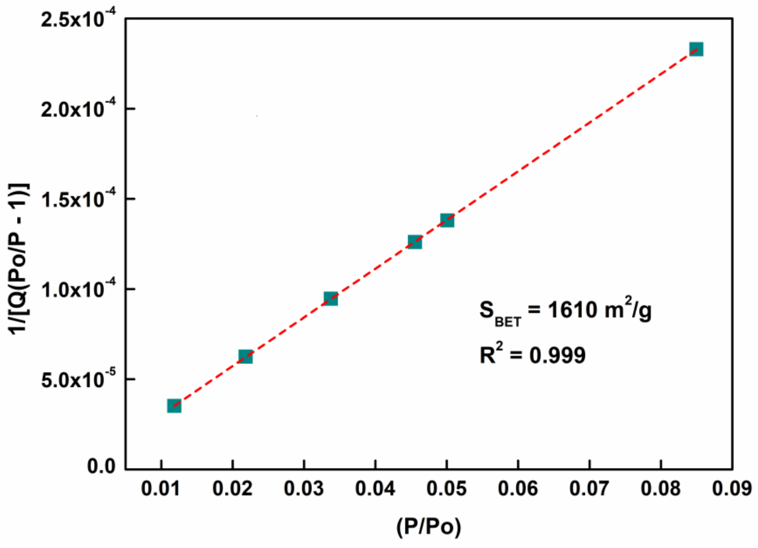

Supplementary Figure 26. BET plot of JUC-642 calculated from N_2 adsorption isotherm at 77 K.

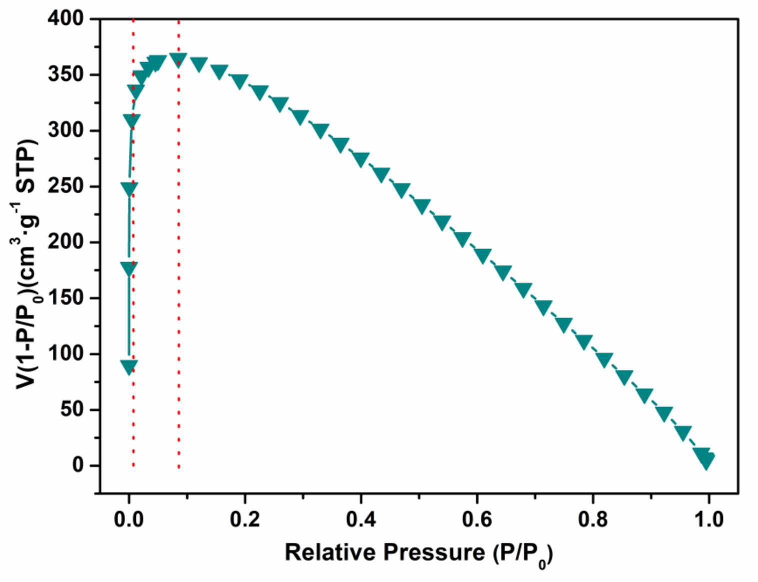

Supplementary Figure 27. Rouquerol BET of JUC-642 calculated from N_2 adsorption isotherm at 77 K.

Supplementary Table 2. Adsorption and selectivity of 3D COFs for benzene and cyclohexane.

3D COF	Surface area (m^2/g)	Bz (cm^3/g)	Cy (cm^3/g)	Ideal Selectivity	Ref
**JUC-641**	**1138**	**162**	**78**	**1.93**	**This work**
**JUC-642**	**1610**	**167**	**77**	**2.02**	
LZU-111	1840	214	164	1.21	16
COF-300-st	1270	221	133	1.54	16
COF-300-rt	39	251	175	1.33	16

Supplementary Table 3. Adsorption and selectivity of POPs for benzene and cyclohexane.

POP	Bz (cm^3/g)	Cy (cm^3/g)	Ideal Selectivity	Breakthrough Selectivity	Ref
**JUC-641**	**162**	**78**	**1.93**	**1.8**	**This work**
**JUC-642**	**167**	**77**	**2.02**	**1.91**	**This work**
CCTF-1	252.1	117.6	1.99	1.74	17
CCTF-2	186.7	82.9	2.09	1.8	17
CCTF-3	39.1	16.3	2.23	1.9	17
PAF-2	39.6	1.9	19.71	-	18
MALP-1	168	131.2	1.19	-	19
MALP-2	156.5	125.9	1.15	-	19
MALP-3	164	130.1	1.17	-	19
MALP-4	160.2	124	1.2	-	19
PAN-1	208.5	140.5	1.38	-	20
PAN-2	198.7	102.1	1.81	-	20
PAN-F	156.2	115.4	1.26	-	21
PAN-T	163.7	138.1	1.26	-	21
CMP-S-1	185.9	100.8	1.71	-	22
MP1	219.7	117.3	1.74	-	23
PCN-AD	281.4	153.1	1.71	-	24
PBI-Ad-1	219.7	142.9	1.83	-	25
PBI-Ad-2	219.7	123.5	1.65	-	25
PCN-TPC	50.5	19.7	2.38	-	26
PCN-TPPC	223.4	28.5	7.27	-	26
UMC-600	190.4	140.5	1.26	-	27
UMC-700	211.1	161.3	1.21	-	27
UMC-800	249.3	195.7	1.18	-	27
FJU-P6	181	83.6	2.01	-	28

FJU-P7	220.7	170.8	1.2	-	28
PCN-TA	289.7	54.9	4.9	-	29
PCN-TC	249	65.1	3.55	-	29
PCN-DC	182.4	34.4	4.92	-	29
POP-1	292.9	175.4	1.55	-	30
MPI-1	344	133.6	2.39	-	31
MPI-2	220	119.5	1.71	-	31
MPI-3	168.3	110.7	1.32	-	31
sPI-1	458.6	226.9	1.88	-	32
sPI-2	505.4	207.5	2.26	-	32

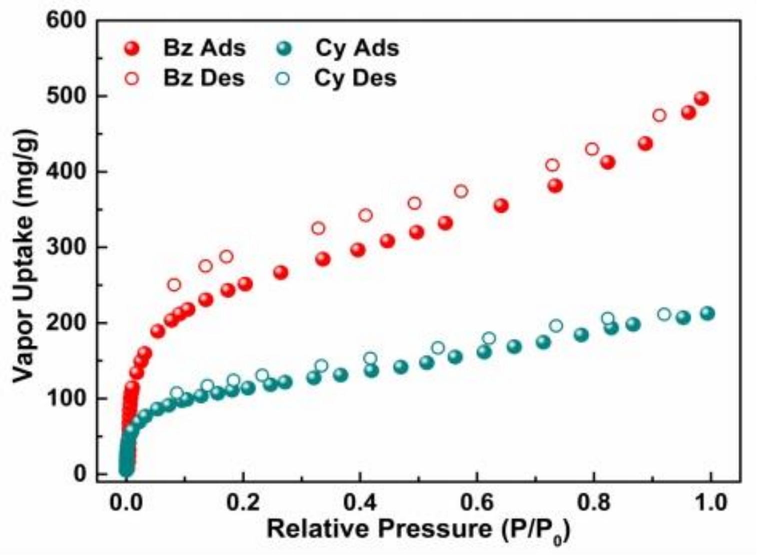

Supplementary Figure 28. Vapor adsorption (filled)–desorption (empty) isotherms of Bz and Cy for JUC-641 at 323 K.

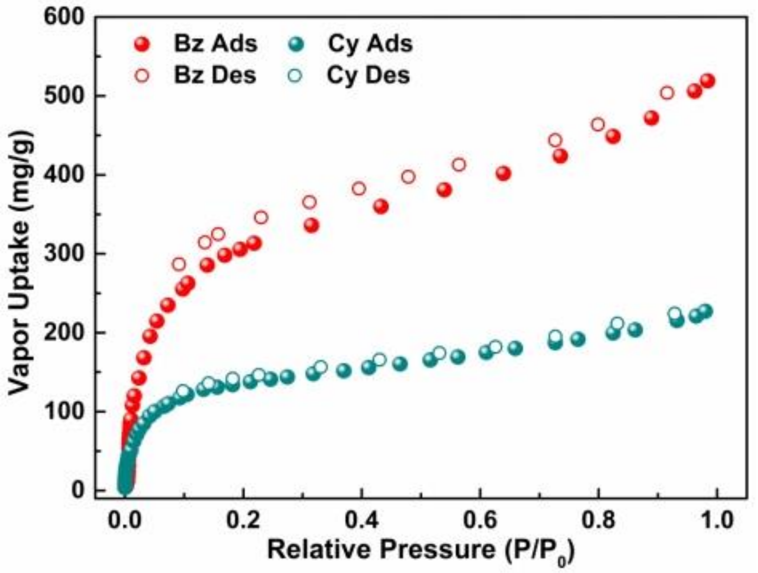

Supplementary Figure 29. Vapor adsorption (filled)–desorption (empty) isotherms of Bz and Cy for JUC-642 at 323 K.

The isosteric heat of adsorption, Q_{st}, defined as

$$-\frac{Q_{st}}{R} = \frac{\partial\,(ln\,P)}{\partial(1/T)}$$

(where P is the pressure, T is the temperature, R is the universal gas constant.)

was determined using the pure component isotherm fits using the virial equation.

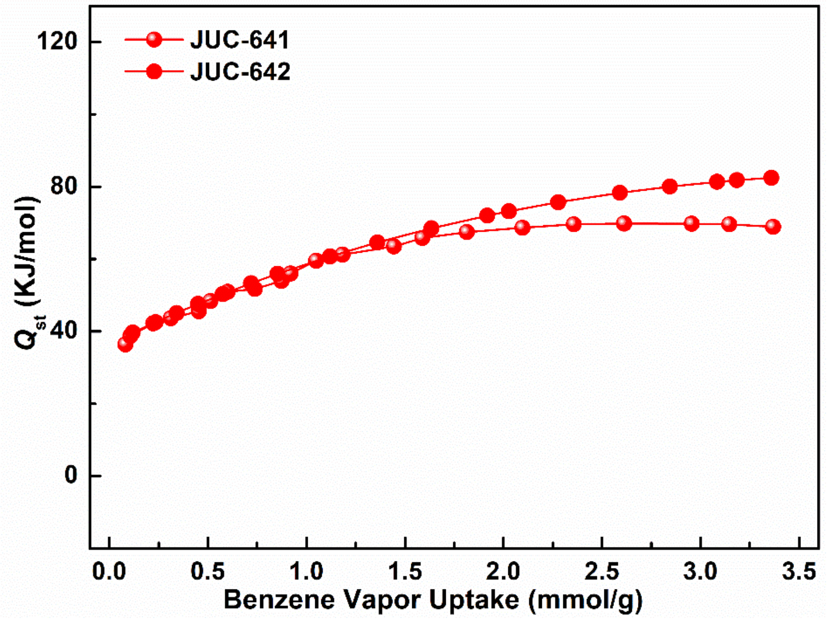

Supplementary Figure 30. Q_{st} of benzene in JUC-641 and JUC-642.

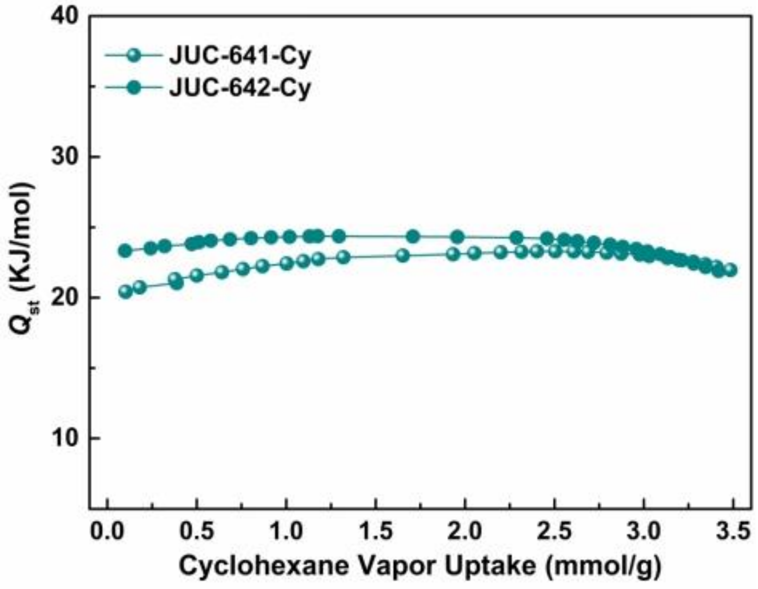

Supplementary Figure 31. Q_{st} of cyclohexane in JUC-641 and JUC-642.

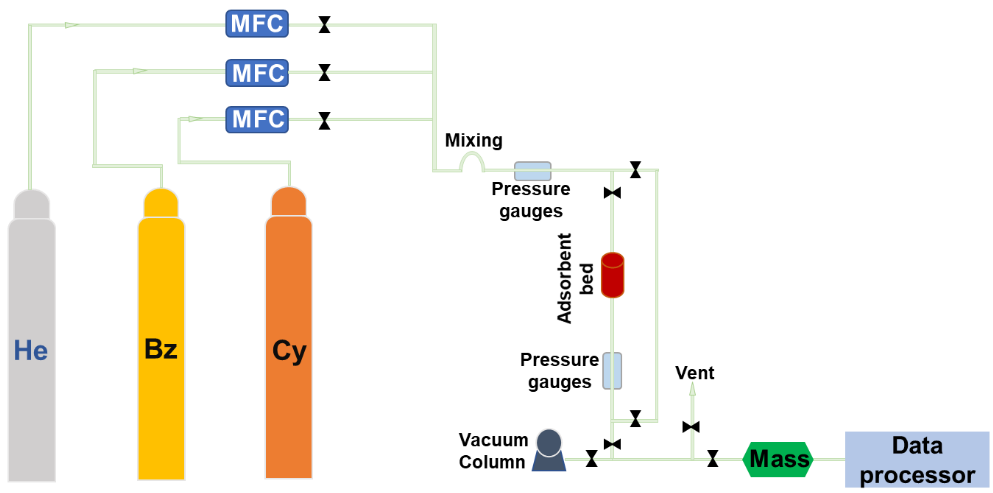

Supplementary Figure 32. Scheme of the dynamic breakthrough apparatus (MFC: Mass flow controller).

The adsorption amount of adsorbent n is calculated according to the equation S1 as:

$$Q_{nad} = Q_{nin} - Q_{nout} = q_{in} \times C_{n0} \times \Delta T - \int_0^t [(q/(1-\sum_1^N C_{nt}))] * C_{nt} dt \quad \text{-------- S1}$$

Q_{nad} is the adsorption amount of adsorbent n.

Q_{nin} is the total flow rate of adsorbent n flowing in of the penetrating column at ΔT.

Q_{nout} is the total flow rate of adsorbent n flowing out of the penetrating column at ΔT.

q_{in} is the total flow rate of gas through the column inlet.

C_{n0} is the total flow rate of gas through the outlet of the column.

ΔT is the total adsorption time (min).

q is the flow rate of carrier gas.

C_{nt} is the percentage concentration of adsorbent N at the entrance of the penetrating column.

The selectivity of gas (1) over gas (2) is calculated according to the equation S2 as:

$$S = (\frac{X_1}{Y_1}) \div (\frac{X_2}{Y_2}) \quad \text{-------- S2}$$

X_1/Y_1: Molar fraction of component 1 in the adsorbed phase/molar fraction of component 1 in the gas phase.
X_2/Y_2: Molar fraction of component 2 in the adsorbed phase/molar fraction of component 2 in the

gas phase.

The diffusion coefficient of vapor is calculated according to the equation S3 as

$$D = \frac{L^2}{6t_{eq}} \quad \text{-------- S3}$$

D is the diffusion coefficient of vapor.

L is the loading length.

t_{eq} is the delay time.

Supplementary Tables 4-6. Multi-constituent Adsorption Breakthrough Result of JUC-641.

Composition	Parameter	Unit	Breakthrough Point	Half Dry Point	Dry Point
Benzene	Time	s	567.3	642.3	850.6
		s/g	1058.3	1198.3	1587.0
	Flow rates the outlet	V/V %	0.107	1.018	1.906
	Adsorption capacity	mmol/g	1.577	1.717	1.729
	Adsorption rate	mmol/g/min	0.157	0.049	0.000
Cyclohexane	Time	s	338.1	350.6	363.1
		s/g	630.8	654.1	677.4
	Flow rates the outlet	V/V %	0.153	1.125	1.982
	Adsorption capacity	mmol/g	0.946	0.964	0.965
	Adsorption rate	mmol/g/min	0.151	0.039	0.000

Composition	Saturated Adsorption Capacity (mmol/g)	Separation rate of Benzene to Cyclohexane
Benzene	1.729	

Cyclohexane	0.965	**1.792**

Composition	Outflow Time	Delay Time	Bed Thickness	Diffusion Coefficient
Benzene	506.85	110.77	1.25	0.0024
Cyclohexane	327.67	16.85	1.25	0.0155

Supplementary Tables 7-9. Multi-constituent Adsorption Breakthrough Result of JUC-642.

Composition	Parameter	Unit	Breakthrough Point	Half Dry Point	Dry Point
Benzene	Time	s	490.2	567.3	694.4
		s/g	922.8	1067.9	1307.2
	Flow rates the outlet	V/V %	0.111	1.017	1.912
	Adsorption capacity	mmol/g	1.376	1.541	1.614
	Adsorption rate	mmol/g/min	0.161	0.086	0.008
Cyclohexane	Time	s	281.8	302.7	315.2
		s/g	530.6	569.8	593.3
	Flow rates the outlet	V/V %	0.123	1.062	2.006
	Adsorption capacity	mmol/g	0.790	0.836	0.846
	Adsorption rate	mmol/g/min	0.162	0.094	0.017

Composition	Saturated Adsorption Capacity (mmol/g)	Separation rate of Benzene to Cyclohexane
Benzene	1.618	

Cyclohexane	0.846	**1.913**

Composition	Outflow Time	Delay Time	Bed Thickness	Diffusion Coefficient
Benzene	519.35	52.72	8	0.2023
Cyclohexane	292.25	7.29	8	1.4622

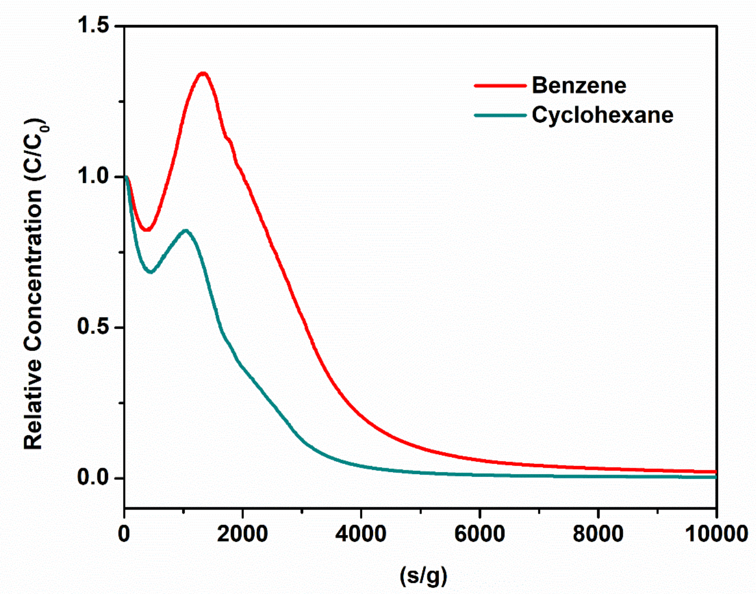

Supplementary Figure 33. Desorption curves for Bz/Cy (v/v, 2/2) mixtures, Ar flow with 50mL·min^{-1}at 373K.

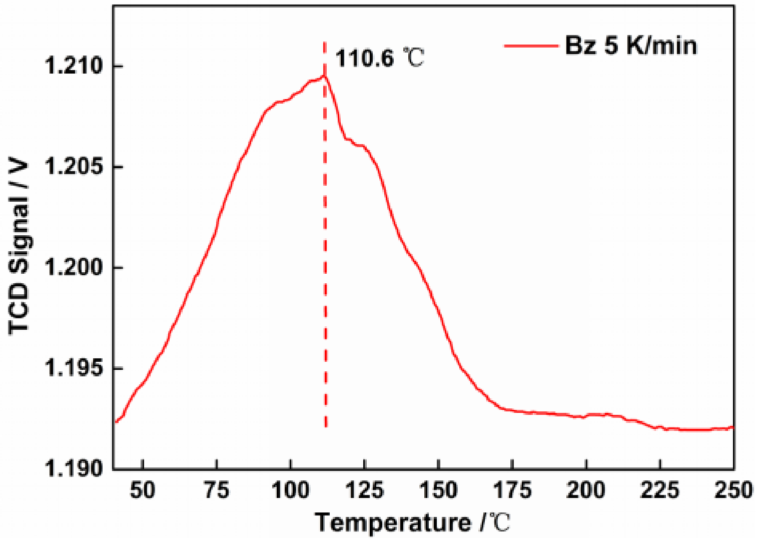

Supplementary Figure 34. TPD of benzene for JUC-642.

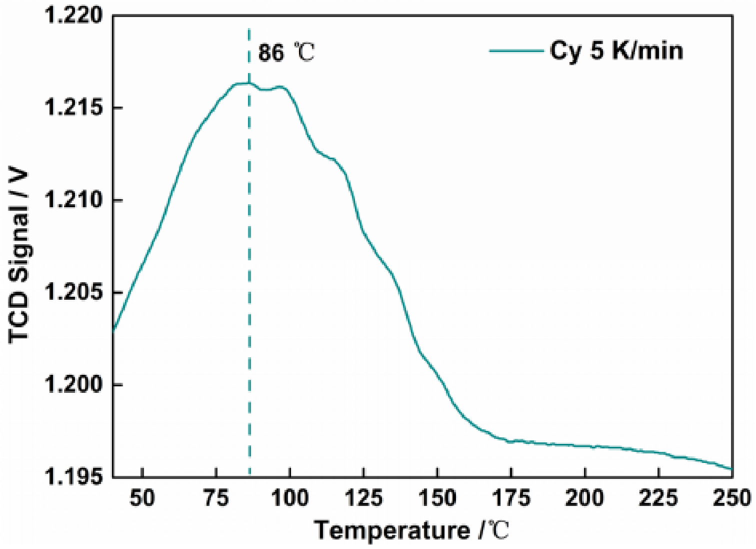

Supplementary Figure 35. TPD of cyclohexane for JUC-642.

Supplementary note 3

All computational calculations were carried using the Gaussian 09 computational packages.[6] Geometry optimization and frequency of the structures were performed using the B3LYP-D3BJ functional, with the dispersion-corrected density functional theory (DFT-D) computational approach to calculate the interaction more accurately, and a general basis set of 6-31g(d,p) was used.[7-13] The optimized geometries were directly used to calculate single-point energy at the M06-2X-D3/def2-TZVP level.[14-15]

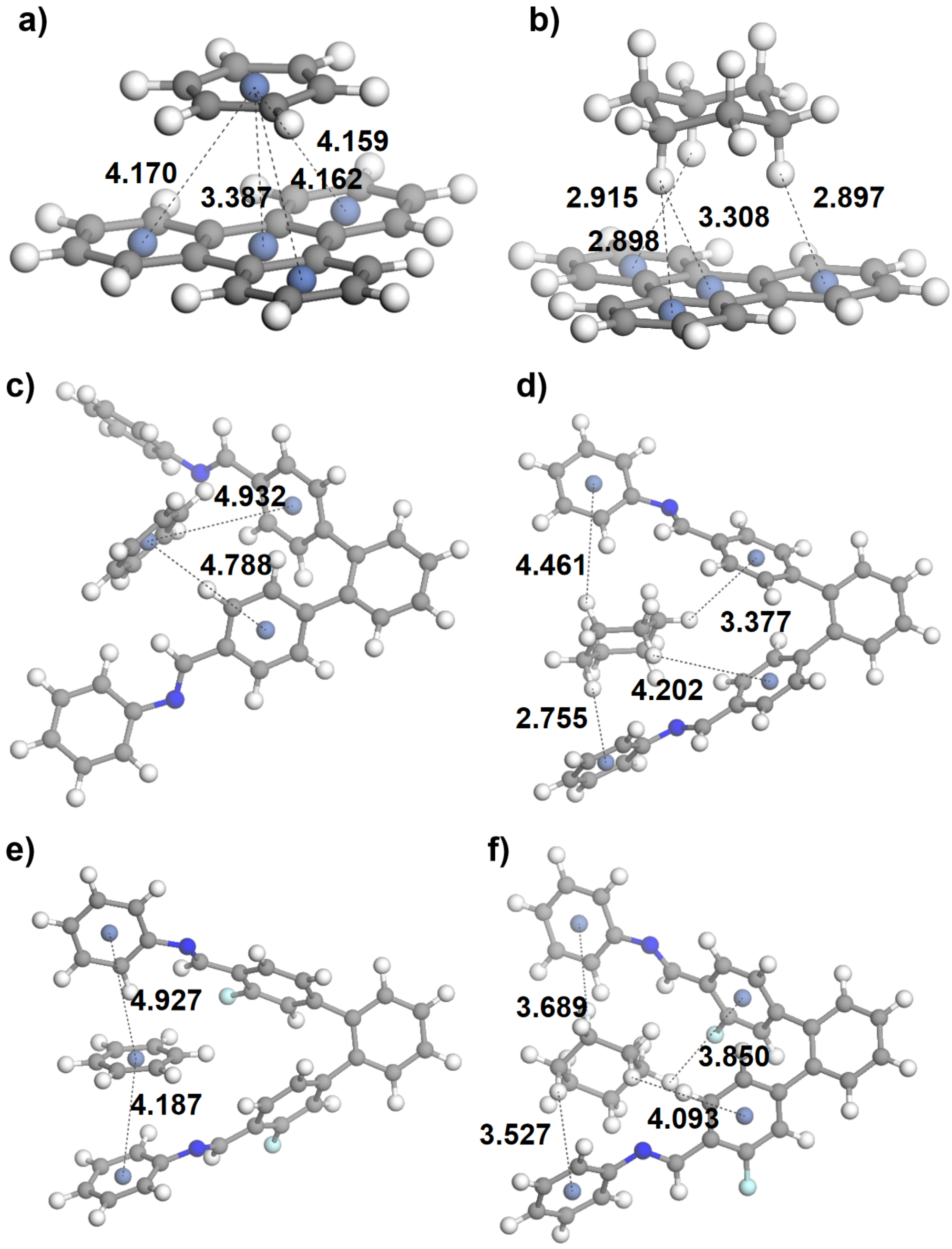

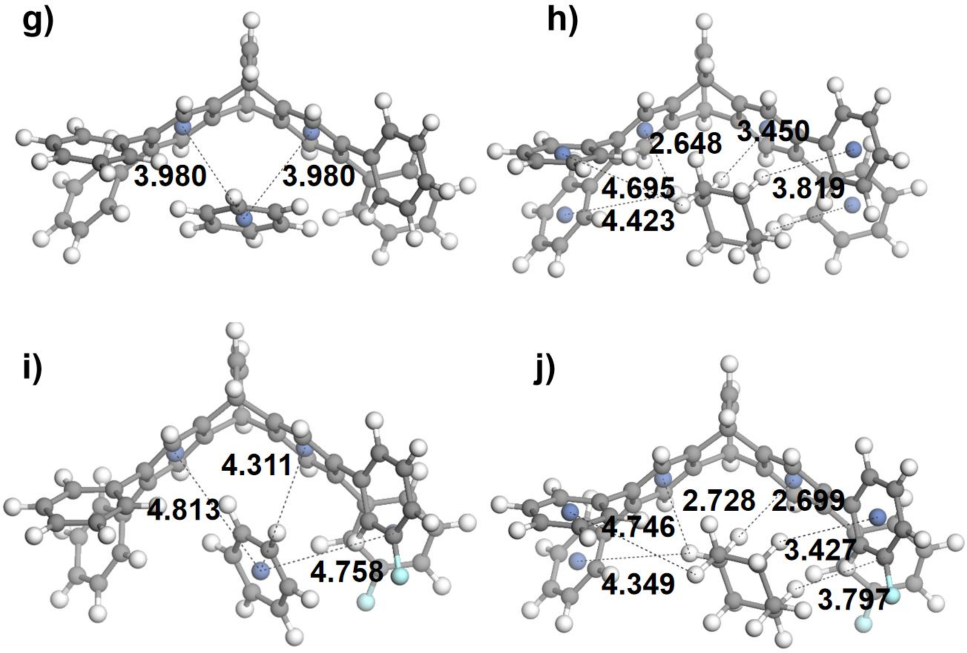

Supplementary Figure 36. C-H···π interaction geometries for trimethylene with (a) benzene and (b) cyclohexane, the angle of the HAPTM in JUC-641 with (c) benzene and (d) cyclohexane, the angle of the HAPTM in JUC-642 with (e) benzene and (f) cyclohexane, the angle of the HFPTP-H in JUC-641 with (g) benzene and (h) cyclohexane, and the angle of the HFPTP-F in JUC-642 with (i) benzene and (j) cyclohexane. Dot lines indicate C−H···π interactions, the unit of distance is Å. (C gray, N blue, H white, F Fluorescent Green, dummy atom: center of the arene ring, violet).

Supplementary Table 10. Unit cell parameters and fractional atomic coordinates for JUC-641 calculated based on the **nia** net.

Space group		*P-62c*	
Calculated unit cell		$a = b = 30.3349$ Å, $c = 22.8400$ Å, $\alpha = \beta = 90°$, $\gamma = 120°$	
Measured unit cell		$a = b = 30.3657$ Å, $c = 22.9435$ Å, $\alpha = \beta = 90°$, $\gamma = 120°$	
Pawley refinement		$Rp = 2.18\%$, Rwp = 2.92%	
atoms	x	y	z
C1	0.19736	0.16681	0.56428
C2	0.24547	0.18954	0.58266
C3	0.27704	0.17461	0.56055
C4	0.25956	0.13676	0.51991
C5	0.2115	0.1143	0.50105
C6	0.17984	0.12897	0.5233
C7	0.35151	0.23719	0.60446
N8	0.32562	0.19439	0.58186
C9	0.42775	0.30002	0.65058
C10	0.47202	0.31456	0.67803
C11	0.48843	0.28164	0.68571
C12	0.46099	0.23451	0.66397
C13	0.41685	0.22	0.63611
C14	0.39955	0.25253	0.62985
C15	0.97517	0.87223	0.50698
C16	0.95036	0.89806	0.50924
C17	0.97433	0.0256	0.50124
C18	0.68025	0.29815	0.71896
C19	0.69231	0.26806	0.68801
C20	0.70428	0.23742	0.71889
H21	0.1734	0.1779	0.58337
H22	0.25717	0.21736	0.61576
H23	0.28323	0.12443	0.5031
H24	0.19891	0.08516	0.46952
H25	0.33806	0.26229	0.60547
H26	0.4152	0.32571	0.64623
H27	0.49307	0.35118	0.69431
H28	0.47329	0.20873	0.66973
H29	0.39585	0.18311	0.62069
H30	0.91252	0.87826	0.52079
H31	0.69256	0.26853	0.64058
C32	0.66667	0.33333	0.69151
H33	0.33333	0.66667	0.35716

Supplementary Table 11. Unit cell parameters and fractional atomic coordinates for JUC-642 calculated based on the **nia** net.

Space group		*P-62c*	
Calculated unit cell		$a = b = 30.0413$ Å, $c = 23.4000$ Å, $\alpha = \beta = 90°$, $\gamma = 120°$	
Measured unit cell		$a = b = 30.1274$ Å, $c = 23.2506$ Å, $\alpha = \beta = 90°$, $\gamma = 120°$	
Pawley refinement		$Rp = 3.96\%$, Rwp = 5.53%	
atoms	x	y	z
C1	-0.80273	0.16755	0.56315
C2	-0.75483	0.19015	0.58226
C3	-0.72289	0.17496	0.56237
C4	-0.73981	0.13703	0.5231
C5	-0.78767	0.11467	0.50358
C6	-0.81973	0.12953	0.5237
C7	-0.64898	0.23721	0.60706
N8	-0.67457	0.19455	0.58438
C9	-0.57259	0.29998	0.65321
C10	-0.5285	0.31395	0.68029
C11	-0.5125	0.28058	0.68786
C12	-0.54022	0.23343	0.66657
C13	-0.58418	0.2194	0.63909
C14	-0.6011	0.25238	0.63271
C15	-0.02487	0.87181	0.50701
C16	-0.04978	0.89773	0.50915
C17	-0.02576	1.02569	0.50122
C18	0.68092	0.29846	0.72004
C19	0.69334	0.26848	0.69016
C20	0.70559	0.2379	0.71996
H21	-0.82701	0.17882	0.58057
H22	-0.74366	0.21803	0.61417
H23	-0.71588	0.1245	0.50793
H24	-0.79982	0.0854	0.47324
H25	-0.66255	0.26232	0.60773
F26	-0.58763	0.33285	0.64767
H27	-0.50714	0.35061	0.69622
H28	-0.52823	0.2073	0.6722
H29	-0.60532	0.18249	0.62391
H30	-0.08771	0.87796	0.52051

H31	0.69346	0.26887	0.64438
C32	0.66667	0.33333	0.69354
H33	0.33333	0.66667	0.35344

Supplementary Table 12. Unit cell parameters and fractional atomic coordinates for JUC-641 calculated based on the **htp** net.

Space group		*Cm*	
Calculated unit cell		a = 64.2467 Å, b = 24.0114 Å, c = 32.7199 Å, $\alpha = \gamma = 90°$, $\beta = 59.5152°$	
atoms	x	y	z
C1	0.63219	0.36105	0.10714
C2	0.65643	0.34524	0.08115
C3	0.51862	0.43104	0.26078
C4	0.54185	0.40843	0.23833
C5	0.73921	0.25048	-0.02995
C6	0.70239	0.2839	-0.03187
C7	0.69379	0.26572	-0.09738
C8	0.68437	0.28191	-0.1258
C9	0.67195	0.33233	-0.11738
C10	0.66968	0.36746	-0.08097
C11	0.67962	0.35196	-0.05328
C12	0.69145	0.3005	-0.06071
C13	0.64306	0.37568	-0.13364
N14	0.66305	0.34848	-0.14792
C15	0.65267	0.3982	-0.2164
C16	0.64589	0.421	-0.24728
C17	0.62216	0.43941	-0.22982
C18	0.60505	0.43309	-0.18155
C19	0.61191	0.41134	-0.15053
C20	0.63581	0.39432	-0.16756
C21	0.60247	0.46908	-0.31782
C22	0.6158	0.47032	-0.26093
C23	0.60918	0.44024	-0.28946
C24	0.77445	0.21175	-0.10188
C25	0.62168	0.70539	0.06444
C26	0.64566	0.72292	0.03831
C27	0.51896	0.57673	0.18508
C28	0.54215	0.59934	0.16381

C29	0.73978	0.74814	0.04494
C30	0.74062	0.74201	0.12282
C31	0.74117	0.68646	0.18735
C32	0.72937	0.66458	0.23329
C33	0.70495	0.67635	0.26478
C34	0.69264	0.71216	0.25007
C35	0.70445	0.73412	0.20416
C36	0.72879	0.72143	0.17239
C37	0.66927	0.64744	-0.65865
N38	0.6934	0.65093	0.31093
C39	0.67471	0.59723	-0.59719
C40	0.66496	0.56893	-0.55396
C41	0.63993	0.5613	-0.52584
C42	0.62469	0.58321	-0.54083
C43	0.63439	0.61169	-0.58392
C44	0.65947	0.61862	-0.61265
C45	0.60648	0.52949	-0.39533
C46	0.62955	0.52957	-0.48112
C47	0.61797	0.55887	-0.43796
C48	0.77601	0.78313	0.04911
C49	0.37123	0.62324	-0.12538
C50	0.34676	0.63618	-0.1052
C51	0.48676	0.56657	-0.20157
C52	0.46272	0.58403	-0.17628
C53	0.26289	0.72836	-0.05405
C54	0.29954	0.69095	-0.12702
C55	0.31064	0.70907	-0.2107
C56	0.3202	0.6919	-0.25764
C57	0.3287	0.63714	-0.27108
C58	0.32774	0.60024	-0.237
C59	0.31845	0.61763	-0.1901
C60	0.30983	0.67223	-0.17668
C61	0.34171	0.65345	0.64214
N62	0.33824	0.61795	-0.31883
C63	0.34022	0.61338	0.57241
C64	0.35081	0.58381	0.52976
C65	0.374	0.56114	0.51114
C66	0.38661	0.56868	0.53533
C67	0.37604	0.59838	0.57797

C68	0.35276	0.62078	0.59684
C69	0.40353	0.53092	0.38107
C70	0.38496	0.52964	0.46629
C71	0.39423	0.55967	0.42363
C72	0.22642	0.76598	-0.05567
C73	0.37976	0.31543	-0.19277
C74	0.35531	0.30206	-0.17074
C75	0.48546	0.42498	-0.27555
C76	0.46151	0.40721	-0.2492
C77	0.26358	0.26897	0.02177
C78	0.26342	0.26753	0.10054
C79	0.26398	0.31482	0.16852
C80	0.27648	0.3335	0.1906
C81	0.30121	0.32129	0.17077
C82	0.31302	0.2902	0.12885
C83	0.30051	0.2713	0.10694
C84	0.27589	0.28344	0.12653
C85	0.30703	0.36087	0.23291
N86	0.31531	0.34315	0.18988
C87	0.31404	0.40403	0.29406
C88	0.32898	0.43032	0.3077
C89	0.35345	0.43919	0.27451
C90	0.363	0.42048	0.22781
C91	0.3481	0.39401	0.21415
C92	0.3234	0.386	0.24712
C93	0.39791	0.46909	0.31394
C94	0.36881	0.47036	0.28803
C95	0.3834	0.44031	0.3009
C96	0.22743	0.23128	0.09662
C97	0.68993	0.28821	0.01795
C98	0.61472	0.33589	0.09922
C99	0.66404	0.30394	0.04626
C100	0.57892	0.37313	0.1675
N101	0.58992	0.35348	0.12455
C102	0.50681	0.4399	0.23539
C103	0.55382	0.39365	0.19008
C104	0.43827	0.47034	0.30209
C105	0.48379	0.47062	0.25711
C106	0.77581	0.22052	-0.0275

C107	0.80005	0.20302	-0.0522
C108	0.31256	0.6886	-0.1031
C109	0.38842	0.64693	-0.16895
C110	0.3386	0.67354	-0.12716
C111	0.42401	0.60995	-0.17085
N112	0.41249	0.63612	-0.19049
C113	0.49845	0.55992	-0.25073
C114	0.4499	0.59632	-0.19949
C115	0.56691	0.52964	-0.32464
C116	0.5215	0.52941	-0.27589
C117	0.22744	0.76007	0.01953
C118	0.20311	0.7774	0.04332
C119	0.59203	0.4441	-0.3463
C120	0.54425	0.43641	-0.30019
C121	0.41335	0.55633	0.33212
C122	0.461	0.56352	0.27947
H123	0.62662	0.39496	0.13518
H124	0.67024	0.36602	0.08826
H125	0.50928	0.44228	0.30002
H126	0.55147	0.40164	0.25914
H127	0.70346	0.22403	-0.10385
H128	0.68679	0.25395	-0.15607
H129	0.6597	0.4088	-0.07391
H130	0.67816	0.38111	-0.02445
H131	0.63105	0.38511	-0.09423
H132	0.67192	0.38267	-0.23077
H133	0.6597	0.42464	-0.28675
H134	0.58547	0.44572	-0.16749
H135	0.59804	0.40734	-0.11113
H136	0.60927	0.39276	-0.28954
H137	0.76442	0.21418	-0.12226
H138	0.60752	0.7247	0.05758
H139	0.65098	0.7577	0.01106
H140	0.50962	0.57129	0.1636
H141	0.55178	0.61228	0.12492
H142	0.76095	0.67595	0.16181
H143	0.73961	0.63687	0.24544
H144	0.67294	0.72338	0.27565
H145	0.6943	0.76257	0.19228

H146	0.65648	0.66718	-0.66904
H147	0.69508	0.60305	-0.62009
H148	0.67738	0.55198	-0.54148
H149	0.60429	0.57784	-0.51785
H150	0.62192	0.62948	-0.59595
H151	0.61793	0.60634	-0.43768
H152	0.79431	0.80491	0.03054
H153	0.37725	0.59329	-0.10633
H154	0.3329	0.61632	-0.07015
H155	0.49685	0.55773	-0.18195
H156	0.45314	0.58856	-0.13608
H157	0.30344	0.75326	-0.19985
H158	0.32113	0.72233	-0.28527
H159	0.33455	0.55571	-0.24742
H160	0.31785	0.58744	-0.16264
H161	0.3235	0.67071	0.65004
H162	0.35416	0.68943	0.63829
H163	0.34348	0.57361	-0.32776
H164	0.32134	0.63161	0.58751
H165	0.3406	0.57804	0.50998
H166	0.40551	0.55058	0.52024
H167	0.38633	0.60449	0.59756
H168	0.39415	0.60715	0.42378
H169	0.23625	0.76341	-0.09602
H170	0.393	0.29727	-0.22908
H171	0.34869	0.27107	-0.18806
H172	0.49478	0.4277	-0.31584
H173	0.45126	0.39548	-0.26794
H174	0.244	0.32502	0.1846
H175	0.26666	0.35872	0.22478
H176	0.33304	0.28008	0.11227
H177	0.3103	0.24581	0.07291
H178	0.28692	0.35689	0.25982
H179	0.29412	0.39712	0.32112
H180	0.32127	0.44463	0.34593
H181	0.38297	0.42682	0.20083
H182	0.35593	0.37878	0.17613
H183	0.38342	0.39283	0.30071
H184	0.20878	0.21121	0.11429

H185	0.58902	0.37434	0.18778
H186	0.81062	0.19965	-0.03291
H187	0.41339	0.59849	-0.13164
H188	0.19296	0.7835	0.08341
H189	0.59196	0.39663	-0.34671
H190	0.54533	0.389	-0.30116
H191	0.4136	0.6038	0.33181
H192	0.45997	0.61094	0.28025

Supplementary Table 13. Unit cell parameters and fractional atomic coordinates for JUC-642 calculated based on the **htp** net.

Space group		*Cm*	
Calculated unit cell		a = 63.9036 Å, b = 23.7884 Å, c = 32.9288 Å,<br>$\alpha = \gamma = 90°, \beta = 58.5211°$	
atoms	x	y	z
C1	0.63007	0.36584	0.11007
C2	0.6548	0.35051	0.08387
C3	0.51325	0.42935	0.26387
C4	0.537	0.40748	0.24154
C5	0.7391	0.25676	-0.02844
C6	0.70262	0.28957	-0.02886
C7	0.69466	0.27002	-0.09446
C8	0.68535	0.28553	-0.12291
C9	0.67245	0.33593	-0.11442
C10	0.66963	0.37181	-0.07794
C11	0.67947	0.35698	-0.05021
C12	0.69179	0.30552	-0.05775
C13	0.64325	0.37808	-0.13074
N14	0.66363	0.35127	-0.14499
C15	0.65378	0.39999	-0.21385
C16	0.64734	0.42197	-0.24525
C17	0.62317	0.43904	-0.22824
C18	0.60532	0.43223	-0.17994
C19	0.61178	0.41127	-0.1483
C20	0.63617	0.39566	-0.1649
C21	0.60407	0.46875	-0.31626
C22	0.61713	0.47005	-0.25957
C23	0.61063	0.43967	-0.28798

C24	0.77506	0.21713	-0.10123
C25	0.62096	0.70391	0.06796
C26	0.64567	0.71986	0.04223
C27	0.51517	0.57659	0.18705
C28	0.53887	0.59844	0.1659
C29	0.73938	0.74135	0.04605
C30	0.74057	0.73595	0.11962
C31	0.74166	0.68033	0.18321
C32	0.73018	0.65935	0.22964
C33	0.70565	0.67246	0.26286
C34	0.69293	0.70876	0.24931
C35	0.70441	0.72978	0.2029
C36	0.72883	0.71554	0.16941
C37	0.67111	0.64514	-0.65922
N38	0.6945	0.64814	0.30962
C39	0.67736	0.59712	-0.5976
C40	0.66752	0.56962	-0.5538
C41	0.64214	0.56172	-0.52463
C42	0.62654	0.58265	-0.53898
C43	0.63624	0.61031	-0.58259
C44	0.66171	0.61742	-0.61261
C45	0.60845	0.52981	-0.39366
C46	0.63182	0.52984	-0.47978
C47	0.6201	0.55945	-0.43647
C48	0.77454	0.77688	0.04829
C49	0.37193	0.62459	-0.13221
C50	0.34712	0.63902	-0.10917
C51	0.48856	0.571	-0.208
C52	0.46461	0.59171	-0.18361
C53	0.26332	0.73432	-0.05295
C54	0.29924	0.69743	-0.12609
C55	0.31275	0.71393	-0.21132
C56	0.32252	0.69455	-0.25789
C57	0.32899	0.6376	-0.26889
C58	0.32536	0.60056	-0.23271
C59	0.31603	0.62013	-0.18634
C60	0.30964	0.67693	-0.17537
C61	0.3515	0.64346	0.6451
N62	0.33795	0.61551	-0.3156

C63	0.34129	0.59247	0.59178
C64	0.34846	0.56633	0.54848
C65	0.37339	0.56204	0.51314
C66	0.39101	0.58777	0.52025
C67	0.38377	0.61582	0.56296
C68	0.35896	0.61693	0.59936
C69	0.39832	0.53123	0.38406
C70	0.38116	0.52999	0.46907
C71	0.38983	0.5602	0.42646
C72	0.2271	0.77177	-0.05282
C73	0.38091	0.30977	-0.19549
C74	0.35613	0.2947	-0.1721
C75	0.48884	0.42663	-0.28179
C76	0.46488	0.40556	-0.25756
C77	0.2636	0.26343	0.02155
C78	0.26335	0.26376	0.09669
C79	0.2632	0.31376	0.16405
C80	0.27534	0.33307	0.18651
C81	0.3003	0.32033	0.1674
C82	0.31271	0.28809	0.12581
C83	0.30056	0.26849	0.10355
C84	0.27571	0.28134	0.12234
C85	0.30507	0.36111	0.2301
N86	0.31409	0.34243	0.18701
C87	0.31114	0.40348	0.29233
C88	0.32588	0.42944	0.30662
C89	0.35085	0.43866	0.27365
C90	0.36111	0.42057	0.22652
C91	0.34643	0.39432	0.21222
C92	0.32121	0.38605	0.24494
C93	0.39411	0.46872	0.31572
C94	0.36591	0.47007	0.28786
C95	0.38006	0.43969	0.30167
C96	0.22881	0.2256	0.09443
C97	0.68937	0.29265	0.02128
C98	0.6129	0.33851	0.10239
C99	0.66288	0.30773	0.04953
C100	0.57567	0.37471	0.17043
N101	0.58753	0.35484	0.12733

C102	0.50192	0.43873	0.23791
C103	0.55001	0.39409	0.19279
C104	0.43437	0.47017	0.30554
C105	0.47869	0.47023	0.25947
C106	0.77595	0.22663	-0.02842
C107	0.80098	0.21051	-0.05512
C108	0.31267	0.69588	-0.10331
C109	0.38907	0.65067	-0.17537
C110	0.33899	0.6796	-0.12882
C111	0.42632	0.62011	-0.17979
N112	0.41466	0.63641	-0.20028
C113	0.50061	0.56086	-0.25701
C114	0.45236	0.60284	-0.20783
C115	0.56825	0.52987	-0.32241
C116	0.524	0.52982	-0.28076
C117	0.22669	0.76618	0.02143
C118	0.20154	0.78141	0.04558
C119	0.59361	0.44342	-0.3444
C120	0.54622	0.44042	-0.30186
C121	0.40909	0.55699	0.3348
C122	0.45644	0.55945	0.28236
H123	0.62457	0.40002	0.1353
H124	0.66777	0.37281	0.08977
H125	0.50402	0.44096	0.30111
H126	0.5455	0.40195	0.2624
H127	0.70418	0.23027	-0.10118
H128	0.68795	0.25793	-0.15134
H129	0.66046	0.41187	-0.07183
H130	0.67769	0.38595	-0.02324
H131	0.63102	0.38734	-0.09322
H132	0.67273	0.38853	-0.22758
H133	0.66135	0.42755	-0.28245
H134	0.5866	0.44546	-0.16647
F135	0.59429	0.40733	-0.10132
H136	0.61001	0.39417	-0.28692
H137	0.76546	0.21576	-0.12011
H138	0.608	0.72498	0.06138
H139	0.65139	0.75341	0.01631
H140	0.50688	0.56961	0.16601

H141	0.54867	0.60833	0.12828
H142	0.76046	0.66839	0.15784
H143	0.7403	0.63184	0.23954
H144	0.67425	0.72156	0.27415
H145	0.69429	0.75738	0.1931
H146	0.65788	0.6604	-0.66764
F147	0.70207	0.60346	-0.62503
H148	0.67972	0.55388	-0.54281
H149	0.60688	0.57669	-0.51677
H150	0.62383	0.62556	-0.5932
H151	0.61952	0.60496	-0.43622
H152	0.79147	0.80018	0.03288
H153	0.37756	0.59245	-0.11663
H154	0.33419	0.61824	-0.07607
H155	0.49734	0.5606	-0.18834
H156	0.4554	0.59771	-0.14546
H157	0.30772	0.75784	-0.20326
H158	0.32445	0.72366	-0.28488
H159	0.32998	0.55646	-0.24063
H160	0.31351	0.59098	-0.15887
H162	0.3583	0.68489	0.64559
H164	0.322	0.59406	0.61922
H165	0.33457	0.54876	0.54276
H166	0.41031	0.58539	0.49304
H167	0.39754	0.63493	0.56827
H168	0.39015	0.60571	0.42638
H169	0.23683	0.77273	-0.09116
H170	0.39388	0.28974	-0.22893
H171	0.3504	0.2626	-0.18756
H172	0.49788	0.43534	-0.31953
F173	0.45377	0.39587	-0.28251
H174	0.24422	0.32539	0.17851
H175	0.26533	0.35945	0.21762
H176	0.33198	0.27842	0.11037
H177	0.31062	0.2437	0.07135
H178	0.28558	0.35867	0.2559
H179	0.2918	0.3976	0.31815
H180	0.31776	0.44341	0.34312
H181	0.38035	0.4278	0.20072

H182	0.35473	0.38138	0.17541
H183	0.37977	0.39418	0.30222
H184	0.2118	0.20251	0.11313
H185	0.58443	0.3769	0.19104
H186	0.81234	0.211	-0.03983
H187	0.41709	0.61931	-0.14129
H188	0.19051	0.78256	0.0839
H189	0.59346	0.39673	-0.34461
H190	0.54632	0.39489	-0.30147
H191	0.40933	0.60367	0.33455
H192	0.45634	0.6048	0.28168

Supplementary References

[1] C, Yu. et al. Three-Dimensional Triptycene-Functionalized Covalent Organic Frameworks with hea Net for Hydrogen Adsorption. *Angew. Chem. Int. Ed.* **61,** e202117101 (2022).

[2] V, Bhalla. et al. A triphenylene based zinc ensemble as an oxidation inhibitor. *Chem. Commun.* **48,** 4722-4724 (2012).

[3] a) T, D. Kühne. et al. CP2K: An electronic structure and molecular dynamics software package - Quickstep: Efficient and accurate electronic structure calculations. *J. Chem. Phys* **152,** 194103 (2020).

b) J, VandeVondele. et al. Quickstep: Fast and accurate density functional calculations using a mixed Gaussian and plane waves approach. *Comput. Phys. Commun.* **167,** 103-128 (2005).

[4] S, Grimme. et al. A consistent and accurate *ab initio* parametrization of density functional dispersion correction (DFT-D) for the 94 elements H-Pu. *J. Chem. Phys.* **132,** 154104 (2010).

[5] a) M, Krack. Pseudopotentials for H to Kr optimized for gradient-corrected exchange-correlation functionals. *Theor. Chem. Acc.* **114,** 145-152 (2005).

b) J, VandeVondele. & J, Hutter. Gaussian basis sets for accurate calculations on molecular systems in gas and condensed phases. *J. Chem. Phys.* **127,** 114105 (2007).

c) C, Adamo. & V, Barone. Toward reliable density functional methods without adjustable parameters: The PBE0 model. *J. Chem. Phys.* **110,** 6158 (1999).

[6] Gaussian 09, Revision A02. 2009, Gaussian Inc., Wallingford CT.

[7] K, Raghavachari. Perspective on "Density functional thermochemistry. III. The role of exact exchange". *Theor. Chem. Acc.* **103,** 361-363 (2000).

[8] Lee. et al. Development of the Colle-Salvetti correlation-energy formula into a functional of the electron density. *Phys. rev., B Condens. Matter.* **37,** 785-789 (1988).

[9] S, H. Vosko. et al. Nusair, Accurate spin-dependent electron liquid correlation energies for local spin density calculations: a critical analysis. *Can. J. Phys.* **58,** 1200 (1980).

[10] P, J. Stephens. et al. Ab Initio Calculation of Vibrational Absorption and Circular

Dichroism Spectra Using Density Functional Force Fields. *J. Phys. Chem. A*. **98,** 11623-11627 (1994).

[11] S, Grimme. et al. Effect of the damping function in dispersion corrected density functional theory. *J. Comput. Chem.* **32,** 1456-1465 (2011).

[12] Robert, H. Cole. Dielectric relaxation and dipole interactions in a high temperature lattice. *Mol. Phys.* **27,** 1-13 (1974).

[13] G, A. Petersson. et al. A complete basis set model chemistry. I. The total energies of closed-shell atoms and hydrides of the first-row elements. *J. Chem. Phys.* **89,** 2193 (1988).

[14] Y, Zhao. & D, G. Truhlar. The M06 suite of density functionals for main group thermochemistry, thermochemical kinetics, noncovalent interactions, excited states, and transition elements: two new functionals and systematic testing of four M06-class functionals and 12 other functionals. *Theor. Chem. Acc.* **120,** 215-241 (2008).

[15] F, Weigend. & R. Ahlrichs. Balanced basis sets of split valence, triple zeta valence and quadruple zeta valence quality for H to Rn: Design and assessment of accuracy. *Phys. Chem. Chem. Phys.* **7,** 3297-3305 (2005).

[16] M, Moroni. et al. Impact of Pore Flexibility in Imine-Linked Covalent Organic Frameworks on Benzene and Cyclohexane Adsorption. *Acs Appl. Mater. & Interfaces.* **14,** 40890-40901 (2022).

[17] J, Yan. et al. Covalent triazine frameworks for the dynamic adsorption/separation of benzene/cyclohexane mixtures. *New J. Chem.* **46,** 7580-7587 (2022).

[18] H, Ren. et al. Targeted synthesis of a 3D porous aromatic framework for selective sorption of benzene. *Chem. Commun.* **46,** 291-293 (2010).

[19] M, Rong. et al. Fabrication of Microporous Aminal-Linked Polymers with Tunable Porosity toward Highly Efficient Adsorption of CO_2, H_2, Organic Vapor, and Volatile Iodine. *Ind. Eng. Chem. Res.* **58,** 17369-17379 (2019).

[20] G, Li. et al. Tetraphenyladamantane-Based Polyaminals for Highly Efficient Captures of CO_2 and Organic Vapors. *Macromolecules.* **47,** 6664-6670 (2014).

[21] G, Li. et al. The cost-effective synthesis of furan- and thienyl-based microporous polyaminals for adsorption of gases and organic vapors. *Chem. Commun.* **52,** 1143-1146 (2016).

[22] T, Chen. et al. Adsorptive Separation of Aromatic Compounds from Alkanes by π–π Interactions in a Carbazole-Based Conjugated Microporous Polymer. *ACS Appl. Mater. Interfaces.* **12,** 56385-56392 (2020).

[23] X, Ma. et al. Pristine and Carboxyl-Functionalized Tetraphenylethylene-Based Ladder Networks for Gas Separation and Volatile Organic Vapor Adsorption. *ACS. Omega.* **3,** 15966-15974 (2018).

[24] C, Shen. et al. Synthesis of 1,3,5,7-tetrakis(4-cyanatophenyl)adamantane and its microporous polycyanurate network for adsorption of organic vapors, hydrogen and carbon dioxide. *Chem. Commun.* **50,** 11238-11241 (2014).

[25] B, Zhang. et al. Tetraphenyladamantane-Based Microporous Polybenzimidazoles for Adsorption of Carbon Dioxide, Hydrogen, and Organic Vapors. *J. Phys. Chem. C.* **119,** 13080-13087 (2015).

[26] G, Deng. & Z, Wang. Triptycene-Based Microporous Cyanate Resins for Adsorption/Separations of Benzene/Cyclohexane and Carbon Dioxide Gas. *ACS Appl. Mater. Interfaces.* **9,** 41618-41627 (2017).

[27] J, Yan. et al. Ultramicroporous Carbons Derived from Semi-Cycloaliphatic Polyimide with Outstanding Adsorption Properties for H_2, CO_2, and Organic Vapors. *J. Phys. Chem. C.* **121,** 22753-22761 (2017).

[28] L, Chen. et al. Microporous polycarbazole frameworks with large conjugated π systems for cyclohexane separation from cyclohexane-containing mixtures. *New J. Chem.* **45,** 22437-22443 (2021).

[29] C, Shen. et al. Synthetic modulation of micro- and mesopores in polycyanurate networks for adsorptions of gases and organic hydrocarbons. *Polym. Chem.* **8,** 1074-1083 (2017).

[30] H, Tan. et al. Selective Adsorption and Separation of Xylene Isomers and Benzene/Cyclohexane with Microporous Organic Polymers POP-1. *ACS Appl. Mater. Interfaces.* **10,** 32717-32725 (2018).

[31] G, Li. & Z, Wang. Microporous Polyimides with Uniform Pores for Adsorption and Separation of CO_2 Gas and Organic Vapors. *Macromolecules* **46,** 3058-3066 (2013).

[32] J, Yan. et al. Monodispersed ultramicroporous semi-cycloaliphatic polyimides for the highly efficient adsorption of CO_2, H_2 and organic vapors. *Polym. Chem.* **2016**, *7*, 7295-7303.
